# Ataxin-2 Regulates *RGS8* Translation in a New BAC-SCA2 Transgenic Mouse Model

**DOI:** 10.1371/journal.pgen.1005182

**Published:** 2015-04-22

**Authors:** Warunee Dansithong, Sharan Paul, Karla P. Figueroa, Marc D. Rinehart, Shaina Wiest, Lance T. Pflieger, Daniel R. Scoles, Stefan M. Pulst

**Affiliations:** Department of Neurology, University of Utah, Salt Lake City, Utah, United States of America; University of Minnesota, UNITED STATES

## Abstract

Spinocerebellar ataxia type 2 (SCA2) is an autosomal dominant disorder with progressive degeneration of cerebellar Purkinje cells (PCs) and other neurons caused by expansion of a glutamine (Q) tract in the ATXN2 protein. We generated BAC transgenic lines in which the full-length human *ATXN2* gene was transcribed using its endogenous regulatory machinery. Mice with the *ATXN2* BAC transgene with an expanded CAG repeat (BAC-Q72) developed a progressive cellular and motor phenotype, whereas BAC mice expressing wild-type human *ATXN2* (BAC-Q22) were indistinguishable from control mice. Expression analysis of laser-capture microdissected (LCM) fractions and regional expression confirmed that the BAC transgene was expressed in PCs and in other neuronal groups such as granule cells (GCs) and neurons in deep cerebellar nuclei as well as in spinal cord. Transcriptome analysis by deep RNA-sequencing revealed that BAC-Q72 mice had progressive changes in steady-state levels of specific mRNAs including *Rgs8*, one of the earliest down-regulated transcripts in the Pcp2-ATXN2[Q127] mouse line. Consistent with LCM analysis, transcriptome changes analyzed by deep RNA-sequencing were not restricted to PCs, but were also seen in transcripts enriched in GCs such as *Neurod1*. BAC-Q72, but not BAC-Q22 mice had reduced *Rgs8* mRNA levels and even more severely reduced steady-state protein levels. Using RNA immunoprecipitation we showed that ATXN2 interacted selectively with *RGS8* mRNA. This interaction was impaired when ATXN2 harbored an expanded polyglutamine. Mutant ATXN2 also reduced RGS8 expression in an *in vitro* coupled translation assay when compared with equal expression of wild-type ATXN2-Q22. Reduced abundance of Rgs8 in Pcp2-ATXN2[Q127] and BAC-Q72 mice supports our observations of a hyper-excitable mGluR1-ITPR1 signaling axis in SCA2, as RGS proteins are linked to attenuating mGluR1 signaling.

## Author Summary

Spinocerebellar ataxia type 2 (SCA2) is an inherited neurodegenerative disorder leading to predominant loss of Purkinje cells in the cerebellum and impairment of motor coordination. The mutation is expansion of a protein domain consisting of a stretch of glutamine amino acids. We generated a mouse model of SCA2 containing the entire human normal or mutant *ATXN2* gene using bacterial artificial chromosome (BAC) technology. Mice expressing a BAC with 72 glutamines (BAC-Q72) developed a progressive cerebellar degeneration and motor impairment in contrast to mice carrying the normal human gene (BAC-Q22). We found that even prior to behavioral onset of disease, the abundance of specific messenger RNAs changed using deep RNA-sequencing. One of the mRNAs with early and significant changes was *Rgs8*. Levels of Rgs8 protein were even further reduced than mRNA levels in BAC-Q72 cerebella suggesting to us that mutant ATXN2 might have a role in mRNA stability and translation. Using a cellular model, we showed that the ATXN2 protein interacted with *RGS8* mRNA and that this interaction differed between normal and mutant ATXN2. Presence of mutant ATXN2 resulted in reduced RGS8 protein translation in a cellular model. Our studies describe a mouse model of SCA2 expressing the entire human *ATXN2* gene and emphasize the role of ATXN2 in mRNA metabolism.

## Introduction

Spinocerebellar ataxia type 2 (SCA2) belongs to the group of neurodegenerative diseases caused by polyglutamine (polyQ) expansion. This group includes SCA1, Machado-Joseph disease (SCA3 or MJD), SCA6, SCA7, SCA17, Huntington's disease, spinal bulbar muscular atrophy (SBMA) and dentatorubral-pallidoluysian atrophy (DRPLA). SCA2 is an autosomal dominant disorder leading to motor incoordination which is caused by progressive degeneration of cerebellar Purkinje cells, and selective loss of neurons within the brainstem and spinal cord [1]. As with most autosomal dominant ataxias, symptoms are characterized by a progressive loss of motor coordination, neuropathies, slurred speech, cognitive impairment and loss of other functional abilities arising from Purkinje cells and deep cerebellar nuclei [2,3].

In SCA2, expansion of a CAG repeat in exon 1 of the *Ataxin-2* (*ATXN2)* gene causes expansion of a polyQ domain in the ATXN2 protein. As in the other polyQ diseases, the length of the polyQ repeat is inversely correlated with age of onset (AO) in SCA2 [1,4]. In contrast to other polyQ diseases, mutant ATXN2 does not enter the nucleus in appreciable amounts in early stages of disease. This is also confirmed by protein interaction studies that have identified ATXN2 interactors with cytoplasmic localization [5–8]. Polyglutamine disorders show their pathology through a toxic gain of function of the protein and larger polyQ expansions have been associated with greater pathology [3,9].

ATXN2 is widely expressed in the mammalian nervous system [1,10,11]. It is involved in regulation of the EGF receptor [12], and the inositol 1,4,5-triphosphate receptor (IP3R) whereby increased cytosolic Ca^2+^ occurs with CAG repeat expansion [13]. ATXN2 functions are also associated with the endoplasmic reticulum [14], and the Golgi complex [15]. Studies in *Caenorhabditis elegans* support a role for ATXN2 in translational regulation as well as embryonic development [6]. *ATXN2* is also important in energy metabolism and weight regulation, as mice lacking *Atxn2*, developed obesity and insulin resistance [16,17]. Furthermore, ATXN2 interacts with multiple RNA binding proteins, including polyA binding protein 1 (PABP1), the RNA splicing factor A2BP1/Fox1, DDX6, TDP-43, and has been localized in polyribosomes and stress granules demonstrating its unique role in RNA metabolism [5,6,8,18].

Several SCA2 mouse models have been generated. We have reported two transgenic mouse models in which expression of full-length *ATXN2* with 58 or 127 CAG repeats (ATXN2-[Q58] or ATXN2-[Q127]) is targeted to Purkinje cells (PCs) using the Purkinje cell protein-2 (*Pcp2*) promoter [19,20]. These lines show progressive motor phenotypes accompanied by the formation of insoluble cytoplasmic aggregates, loss of PCs, and shrinkage of the molecular layer associated with the reduction of calbindin staining in PC bodies and dendrites. Onset of the motor phenotype of Pcp2-ATXN2[Q127] mice is associated with reduced PC firing that is progressive with age [20]. Another Atxn2-CAG42 knock-in mouse model demonstrated very late-onset motor incoordination associated, but this was seen only in homozygous knock-in animals. This was associated with Pabpc1 deficiency, and upregulation of Fbxw8, but without loss of calbindin staining or downregulation of *Calb1* mRNA [21].

In order to model human diseases using cis-regulatory elements, recent mouse and rat models have been created by transgenesis using human bacterial artificial chromosomes (BACs) [22–27]. In the BAC approach, an entire human gene including introns and regulatory regions is introduced into the mouse genome. BAC models often have lower genomic copy numbers than conventional cDNA transgenic models resulting in more physiological expression levels and a potentially more faithful late onset of disease.

We developed new BAC-SCA2 transgenic mouse lines expressing full-length human wild-type or mutant *ATXN2* genes including upstream and downstream regulatory sequences. BAC mice with mutant *ATXN2* exhibited progressive neurological symptoms and morphological changes in cerebellum. We used this mouse model to confirm changes in key PC-genes identified in a cDNA transgenic model, in particular the effects of mutant ATXN2 on Rgs8 steady state protein levels.

## Results

### Generation and characterization of BAC-SCA2 mice

**Fig 1:**
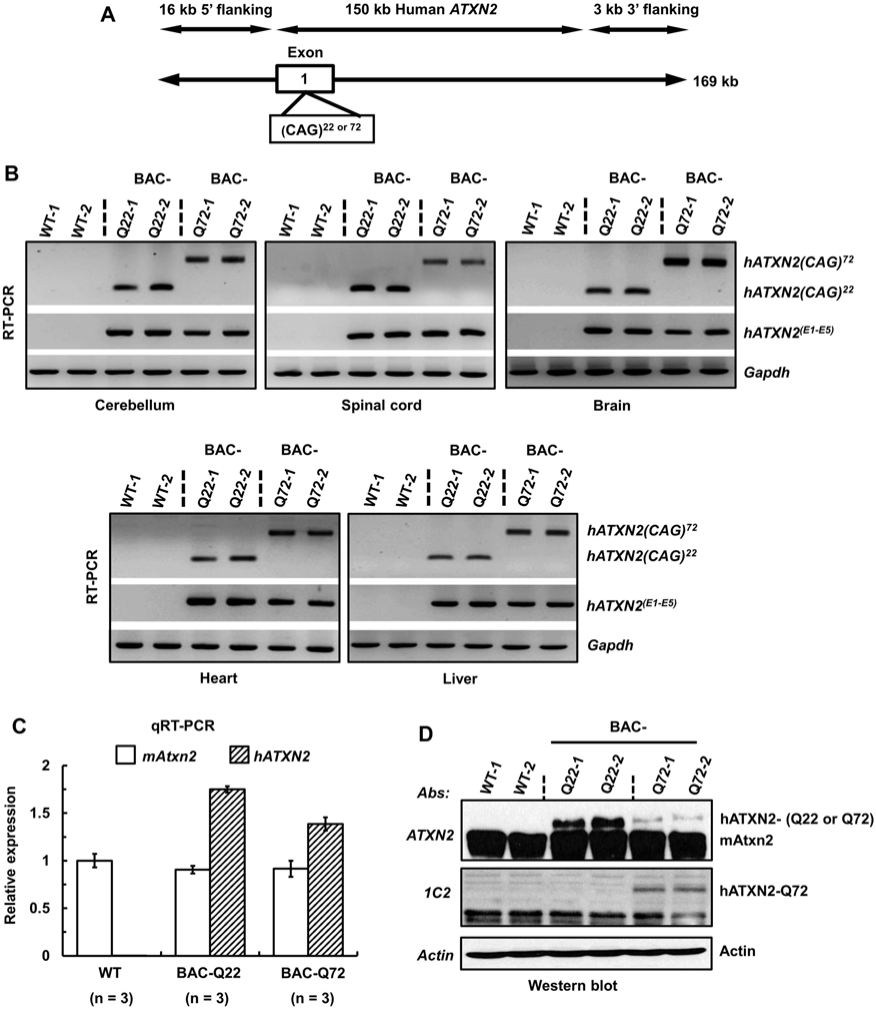
Generation of a BAC-SCA2 transgenic mouse model. **(A)** Schematic representation of the modified 169 kb BAC containing the entire 150 kb human *ATXN2* genomic locus, plus 16 kb 5’-flanking and 3 kb 3-’ flanking genomic regions. For the BAC-Q72 line, the BAC was engineered to replace the endogenous *ATXN2* exon-1 CAG22 with CAG72 repeats. **(B)** RT-PCR analyses revealed expression of BAC-Q22 or BAC-Q72 in mouse CNS and non-CNS tissues. Synthesized cDNAs from mouse tissues were subjected to RT-PCR analysis using human *ATXN2* specific primers and CAG primers as indicated. The *Gapdh* gene was amplified as an internal control. **(C)** The BAC-Q22 transgene is expressed at higher levels than the BAC-Q72 transgene. Quantitative RT-PCR analyses of cerebellar RNA from wild-type and transgenic mice measuring endogenous murine and human *ATXN2* transgene. Note that direct comparison with expression levels of murine *ATXN2* is not possible owing to different primer sets. Three animals per group were used for these analyses. **(D)** Western blot analyses of human ATXN2 protein in BAC-Q22 or BAC-Q72 mouse cerebella. Protein extracts from wild-type and transgenic mouse cerebella were subjected to Western blot analyses using ATXN2 or 1C2 mAbs. Two animals per group were used for Western blot analyses. Representative Western blots of three independent experiments are shown. β-actin was used as loading control.

To understand the pathological and behavioral effects in the context of physiologic expression of human wild-type and mutant *ATXN2*, we engineered a 169 kb human BAC (RP11-798L5) that contained the entire 150 kb human *ATXN2* locus with 16 kb of the 5’ flanking genomic sequence and 3 kb of the 3’ flanking genomic sequence (Fig 1A). The authenticities of these constructs were subsequently verified by Southern blot and restriction site analyses (S1 Fig). The CAG tract was mutation-free when sequenced from both strands. After transgenic microinjection of purified intact BAC DNAs, one line each for control (BAC-*ATXN2*-Q22) and one for mutant mice (BAC-*ATXN2*-Q72) was further analyzed. These lines will be designated as BAC-Q22 and BAC-Q72 in the remainder of the text. Quantitative PCR (qPCR) analyses of genomic DNA revealed that both BAC-Q22 and BAC-Q72 mice had tandem integrates of 10 and 4 copies of the *ATXN2* transgene, respectively. In RT-PCR analyses, both BAC-Q22 and BAC-Q72 mice demonstrated the expression of intact human *ATXN2* transcripts throughout the central nervous system (CNS), including cerebral hemispheres, cerebellum and spinal cord (Fig 1B). Non-CNS tissues, including heart and liver also showed *ATXN2* transgene expression (Fig 1B). The authenticities of PCR products were confirmed by sequencing. We further determined relative expression of *ATXN2* transcripts in the two BAC transgenic lines by quantitative RT-PCR. BAC-Q22 cerebella had higher expression of human *ATXN2* than BAC-Q72 cerebella while the expression of endogenous mouse *Atxn2* remained unchanged in both compared with wild-type mice (Fig 1C). To assess protein expression, we performed Western blot analysis using cerebellar extracts of 16 week-old animals and a monoclonal antibody (mAb) to human ATXN2. The results showed that BAC mice expressed full-length human wild-type or mutant ATXN2 protein. Of note, protein levels of ATXN2-Q22 were higher than those of ATXN2-Q72. Furthermore, we confirmed the ATXN2-Q72 protein expression using 1C2 mAb, an antibody against an expanded polyQ epitope in Western blot analyses (Fig 1D). These results demonstrate that human *ATXN2* transgenes (*ATXN2*-Q22 and *ATXN2*-Q72) were properly expressed in BAC mice.

In addition to *ATXN2*, three overlapping genes (*U7*.*1–202* snRNA, *RP11-686G8*.*1–001* and *RP11-686G8*.*2–001*) are contained in the human BAC. Quantitative RT-PCR analyses of wild-type and BAC transgenic mouse cerebellar RNAs demonstrated that the relative expression of each overlapping gene to that of the *ATXN2* transgene did not differ between BAC-Q22 and BAC-Q72 animals indicating these genes did not contribute to the phenotypes associated with CAG expansion in the *ATXN2* gene (S2 Fig).

### *ATXN2* transgene expression parallels endogenous *Atxn2* expression *in vivo*

The Allen Brain Atlas shows widespread expression of human *ATXN2* with very significant expression levels in the cerebellum [28]. Given the nature of *ATXN2* expression in brain, we determined the expression of human *ATXN2* transgene transcript in sub-regions of mouse brain including spinal cord using qRT-PCR. Expression of endogenous *mAtxn2* was evident in many regions including frontal, occipital and olfactory cortex, hippocampus, thalamus, basal ganglia, cerebellum and spinal cord. Human *ATXN2* transgene expression was found in all regions tested, but relatively higher expression was observed in the basal ganglia (S3 Fig).

**Fig 2:**
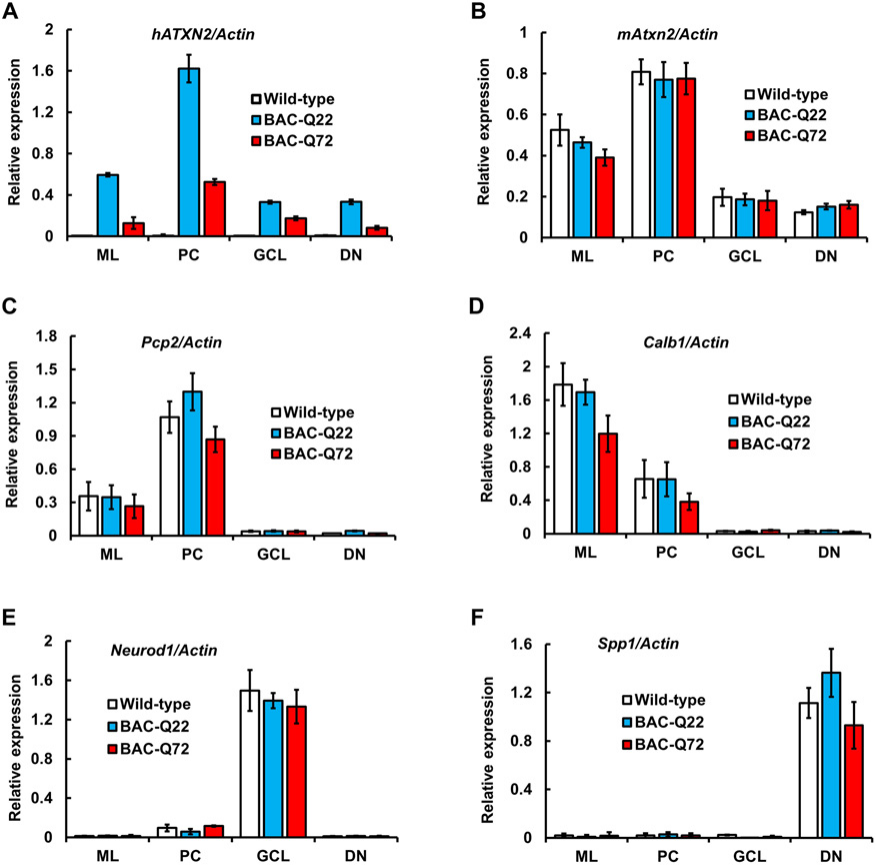
BAC derived- *hATXN2* mRNA is identified in multiple layers of the cerebellum and deep cerebellar nuclei. Expression of transgenic h*ATXN2* and murine *Atxn2* mRNAs in cerebellar fractions isolated by Laser Capture Microdissection (LCM): ML, molecular layer; PC, Purkinje cell layer; GCL, granule cell layer; DN, dentate nucleus. **(A-B)** Quantitative RT-PCR analyses of transgenic *hATXN2* (A) and endogenous *mAtxn2* (B). **(C-F)** Relative enrichment of cell-type specific marker genes; *Pcp2*, *Calb1*, *Neurod1* and *Spp1* for each fraction as determined by qRT-PCR. The error bars indicate ± SD.

As cerebellar degeneration is predominant in SCA2, we further examined the expression patterns of the *ATXN2* transgene in discrete areas of the cerebellum using laser-capture microdissection (LCM). We captured molecular layer (ML), Purkinje cells (PCs), granule cell layer (GCL) and dentate nuclear (DN) fractions. Relative enrichment was determined by measuring expression of a cell-type specific marker genes using qRT-PCR. Evidence for expression of endogenous *mAtxn2* was found in all fractions, but was highest in Purkinje cells. Expression of transgenic *ATXN2* was also seen in all fractions, although small differences in expression levels existed between BAC-Q22 and BAC-Q72 (Fig 2A and 2B). LCM was remarkably successful in separating cerebellar neuronal population as shown by expression of marker genes for PCs and molecular layer (*Pcp2 and Calb1*), granule cells (*Neurod1*) and dentate neurons (*Spp1*) (Fig 2C and 2F). In summary, inclusion of regulatory regions in the human BAC transgene led to expression of the transgene that mirrored expression of mouse *Atxn2* including low but detectable expression in GCs and DNs.

### Phenotypic analyses of BAC transgenic mice

By visual inspection both BAC transgenic lines (BAC-Q22 and BAC-Q72) had a smaller body size than wild-type littermates beginning at 8 weeks of age. By 24 weeks of age, both BAC transgenic mice weighed about 30% less than their wild-type littermates (Wild-type = 33.9 ±3.8; BAC-Q22 = 24.6 ±3.6 and Wild-type = 32.1 ±2.8; BAC-Q72 = 22.9 ±3.7).

**Fig 3:**
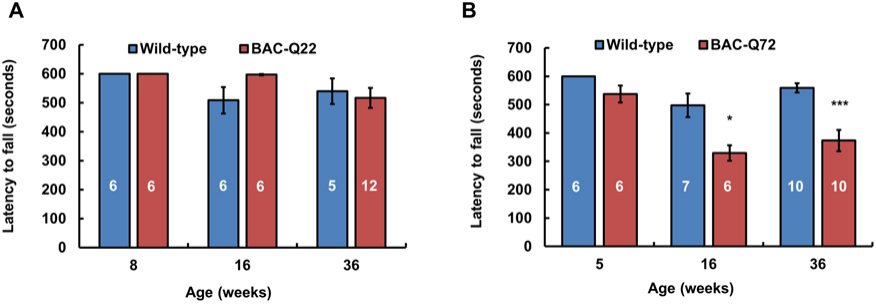
Motor phenotype of *ATXN2* BAC transgenic mice on the accelerating rotarod. **(A)** BAC-Q22 mice performed as well as wild-type mice at all ages. **(B)** BAC-Q72 mice performed significantly worse than wild-type littermates on the rotarod starting at 16 weeks of age. Data represent mean ± SEM of three trials on the test day (day 3). Number of animals tested are shown within the bars. Significance was determined using repeated measures ANOVA with post-hoc test correction. *p<0.05 and ***p<0.001.

BAC-Q72 mice did not show an abnormal home cage behavior. To assess the development of motor impairment, both BAC transgenic lines and wild-type littermates were tested using the accelerating rotarod paradigm at several time points (Fig 3). BAC-Q22 mice performed as well as wild-type littermates at 8, 16 and 36 weeks of age (Fig 3) suggesting that expression of wild-type human *ATXN2* was not detrimental to motor function.

BAC-Q72 mice were tested at 5, 16 and 36 weeks of age and compared with their wild-type littermates. BAC-Q72 mice showed normal performance at 5 weeks (Fig 3) and at 12 weeks (S4A Fig). Of note, testing at 12 weeks was performed on mice housed under slightly different conditions, which may explain the relatively poor performance of wild-type mice. At 16 weeks of age, performance of BAC-Q72 mice became significantly worse than wild-type mice (Fig 3; p<0.05) and mice continued to perform poorly as they aged (24 and 36 weeks old, S4A Fig and Fig 3). Taken together, these results indicate that BAC-Q72 transgenic mice develop a progressive age-dependent motor impairment.

### Cerebellar morphological changes in BAC-Q72 mice

**Fig 4:**
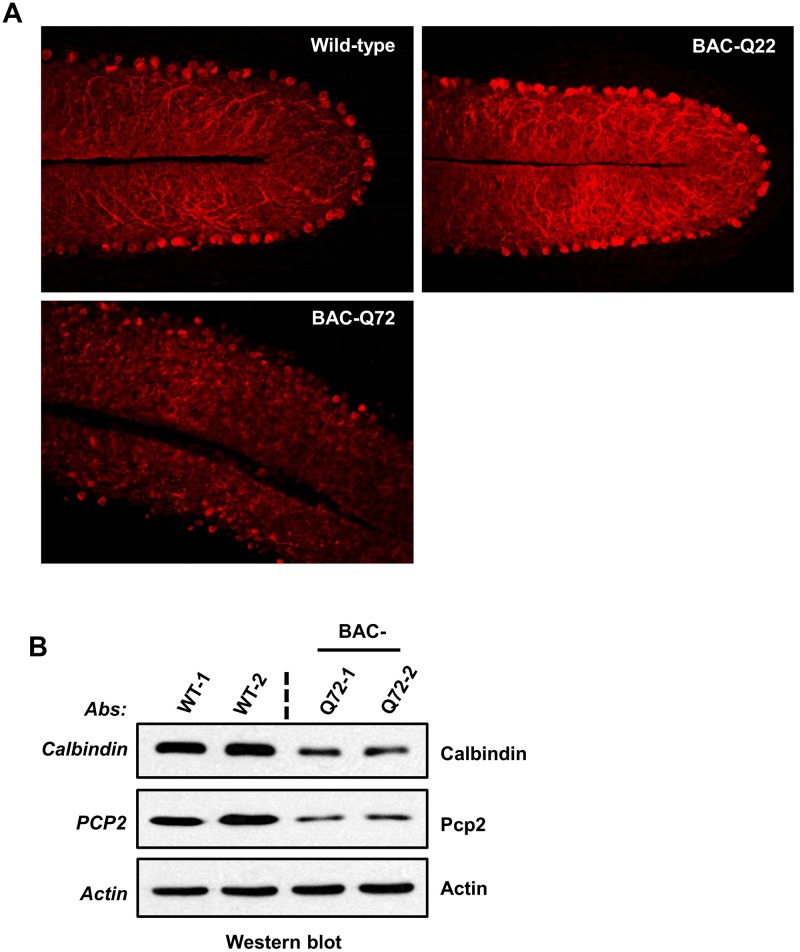
PC morphology in BAC-Q22 and BAC-Q72 mice. **(A)** Representative micrographs of calbindin-28k immunostaining of PCs in the cerebellum of BAC-Q22, BAC-Q72, and wild-type mice at 24 weeks of age. Note that reduction of calbindin immunoreactivity and disorganization of the PC layer are only observed in BAC-Q72 cerebella (see also S4B Fig). **(B)** Western blot analyses show reduction of Calb1 and Pcp2 protein in BAC-Q72 mouse cerebella compared with wild-type at 24 weeks of age. Two animals per group were used for these analyses and the blots represent three independent experiments.

To investigate morphological changes associated with the expression of mutant ATXN2 protein, we compared cerebellar sections from BAC transgenic lines with wild-type mice. Immunostaining with calbindin-28k antibody revealed PC morphological changes in BAC-Q72 mice at 24 weeks of age, but not in BAC-Q22 or wild-type mice (Fig 4A). To more quantitatively assess this change, we performed Western blotting and verified reduction of Calb1 and Pcp2 proteins in BAC-Q72 mouse cerebella (Fig 4B). As observed in the Pcp2-ATXN2[Q127] model, cerebellar morphology was still normal at a time when key mRNA transcripts had already declined. Thus, calbindin-stained cerebellar sections and PC counts of BAC-Q72 mice at 12 weeks showed normal cerebellar morphology and unaltered PC counts [18.8 ±1.2 in WT, n = 3 animals, and 19.4 ±1.1 in BAC-Q72 mice, n = 3 animals, p = 0.51] (S4B, S4C Fig).

### Cerebellar gene expression changes in BAC-Q72 mice

We previously reported that steady-state mRNA levels of specific PC transcripts preceded behavioral onset in an SCA2 model targeting transgene expression to PCs [20]. Expression changes in these genes (*Calb1*, *Pcp2*, *Grid2* and *Grm1*) also preceded the onset of a decrease in PC firing. Expression changes were progressive over time and paralleled deterioration of motor behavior.

**Fig 5:**
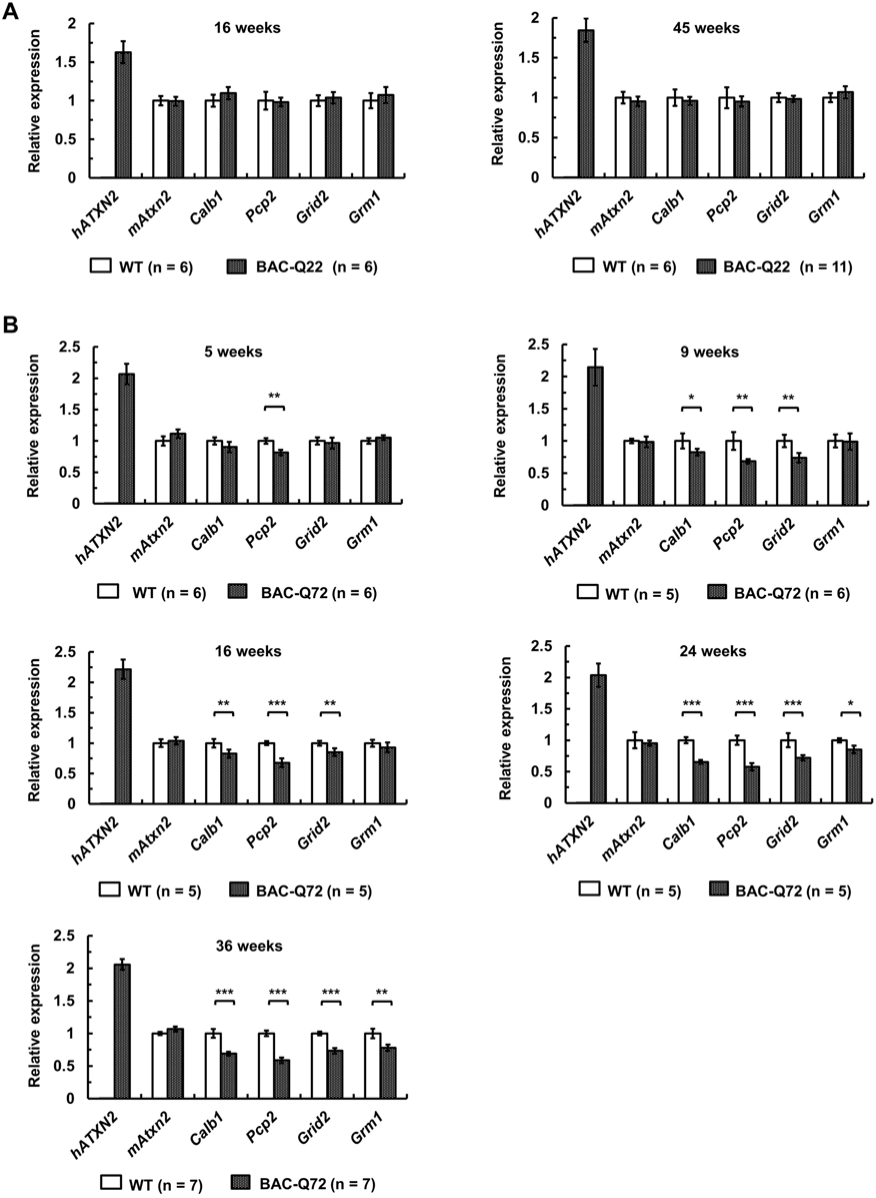
Early expression changes of key cerebellar genes including several PC-specific genes measured by quantitative RT-PCR. **(A)** No significant changes in BAC-Q22 mice compared with wild-type at 16 and 45 weeks of age. **(B)** In BAC-Q72 mice, a small reduction of *Pcp2* mRNA levels is seen at 5 weeks, but significant reductions in three genes are only seen at 9 weeks. Reductions in expression of *Grm1* occur late (weeks 24 and 36). Of note, mRNA levels of mouse *Atxn2* remain unchanged throughout. Genes tested: human transgene (h*ATXN2*), mouse Ataxin-2 (m*Atxn2*), calbindin 28-kDa (*Calb1*), PC protein 2 (*Pcp2*), glutamate receptor ionotropic delta-2 (*Grid2*) and metabotropic glutamate receptor 1 (*Grm1*). n: animal numbers for each genotype and age group are listed in brackets. Gene expression was normalized to beta-actin. Student’s two-tailed t-test compared expression in BAC transgenic mice with wild-type mice in each age group. *p<0.05, **p<0.01, ***p<0.001. Error bars represent ± SD.

To investigate whether similar changes occurred in BAC transgenic mice as we previously observed in Pcp2-ATXN2[Q127], we performed qRT-PCR to measure transcript levels of PC-specific genes at different ages. At 16 and 45 weeks, BAC-Q22 mice were indistinguishable from wild-type mice including expression of endogenous mouse *Atxn2* (Fig 5A).

In BAC-Q72 mice, however, expression of *Pcp2* showed significant reductions (p<0.01) as early as 5 weeks. All other genes tested remained unchanged compared to wild-type (Fig 5B). At 9 and 16 weeks of age, significant reductions in *Calb1* (p<0.05) and *Grid2* (p<0.01) were seen and were progressive (Fig 5B). Steady-state levels of *Grm1* decreased only at 24 weeks (p<0.05). Endogenous mouse *Atxn2* expression levels did not change in BAC-Q72 mice at any time point when compared with wild-type. Taken together, these data demonstrated that a subset of PC-enriched genes showed a progressive reduction in steady-state mRNA levels in BAC-Q72 mice, whereas they remained unchanged in BAC-Q22 animals.

### Cerebellar transcriptional changes in BAC-Q72 mice

To further characterize the BAC-Q72 line and compare it with the well-characterized Pcp2-ATXN2[Q127] line, we performed transcriptome analysis by deep RNA-sequencing of cerebellar RNA. We chose time points for both lines just prior to behavioral and morphological changes, i.e. 8 weeks for the BAC-Q72 line and 6 weeks for the Pcp2-ATXN2[Q127] line. For both sets of RNAs, quality of reads and alignments were high (see methods).

**Fig 6:**
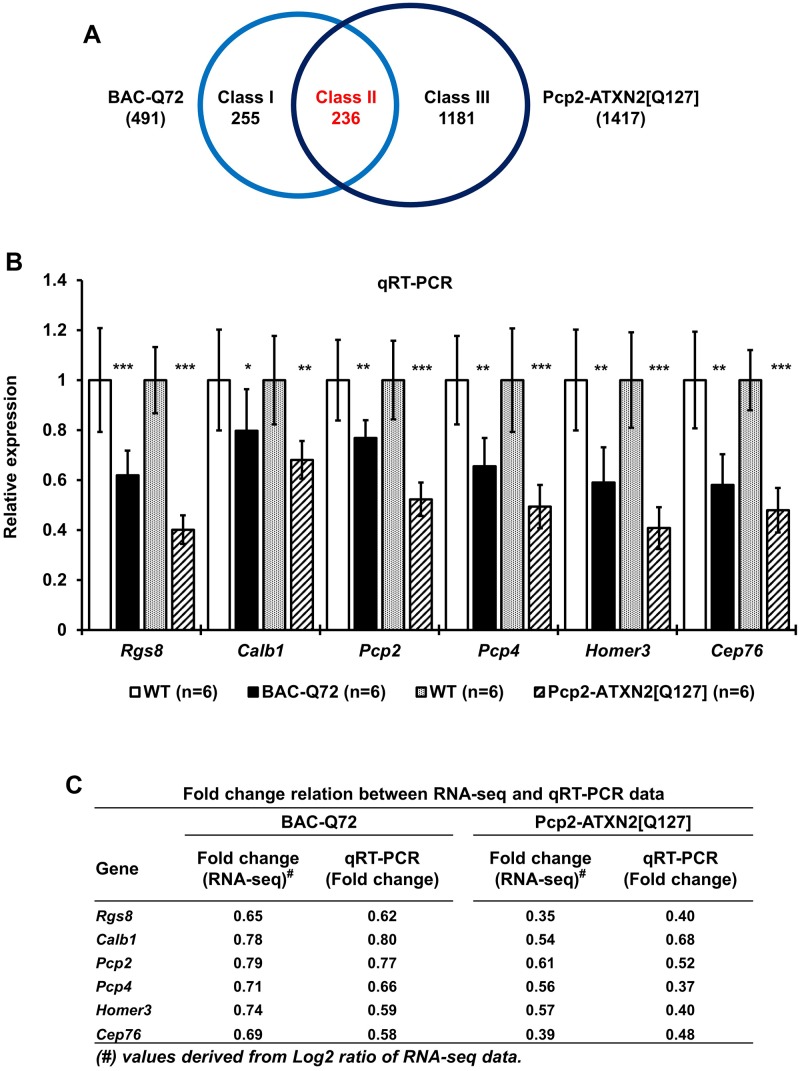
Comparison of transcriptome changes in BAC-Q72 and Pcp2-ATXN2[Q127] mice. **(A)** The Venn diagram of transcriptome changes using an FDR ≥15 and Log2 ratio of change ≥|0.3|. Class I transcripts are changed only in BAC-Q72 and class III transcripts changed only in Pcp2-ATXN2[Q127] cerebella. A total of 236 transcripts (class II) are significantly altered in both models. **(B)** Validation of six overlapping genes (class II) by qRT-PCR. Cerebellar RNAs from BAC-Q72 and WT littermates (both at 8 weeks of age) and Pcp2-ATXN2[Q127] and WT littermates (both at 6 weeks of age) show significant reductions of transcript expression. Genes tested are; *Rgs8*, *Calb1*, *Pcp2*, Purkinje cell protein 4 (*Pcp4*), Homer homolog 3 (Drosophila) (*Homer3*) and Centrosomal protein 76 (*Cep76*). Gene expression levels were normalized to beta-actin. Six animals from each group were used in this experiment. Data are means ± SD, *p<0.05 **p<0.01, ***p<0.001, Student t-test. **(C)** Fold change relation between RNA-seq data and observed experimental qRT-PCR data are tabulated.

We observed significant changes of 1417 transcripts in Pcp2-ATXN2[Q127] and 491 transcripts in BAC-Q72 mice with a false discovery rate (FDR) of ≥15 and a log2 ratio of change ≥|0.30| (Fig 6A). With these filtering parameters, 255 transcripts were only seen in the BAC-Q72 line (class I), 236 transcripts were shared between the two lines (class II) and 1181 transcripts were changed only in the Pcp2-ATXN2[Q127] line (Class III). We validated changes in several of the class II transcripts by qRT-PCR using cerebellar RNA samples from BAC-Q72 mice (8 weeks old) and Pcp2-ATXN2[Q127] (6 weeks old), and compared with their respective WT littermates (Fig 6B). The concordance between RNA-seq and qRT-PCR was high (Fig 6C).

The top 50 transcripts changed in the BAC-Q72 line are shown in S1 Table and the top 50 transcripts changed in the Pcp2-ATXN2[Q127] line are presented in S2 Table. This table also shows that most of these transcripts are changed in the BAC-Q72 line as well, although with a smaller degree of change or a lower FDR. S3 Table lists the top class II genes sorted by FDR in the BAC-Q72 line. This represents a subset of the 236 overlapping genes shown in Fig 6A.

In order to gain insight into the molecular function of altered transcripts in BAC-Q72 and Pcp2-ATXN2[Q127] mice, we performed Gene Ontology (GO) analysis. This is shown in S4 Table and indicates that many of the significant GO terms are shared by the two models. Of note, GO terms relate to known functions of PC such as calcium homeostasis, glutamate-mediated signaling and voltage-gated ion channels. In summary, these data indicate a significant overlap of altered transcripts and shared functions in both SCA2 models at comparable stages just prior to onset of morphological and behavioral changes.

We were also interested in the nature and expression pattern of transcripts in class I and class III (Fig 6). We confirmed changes in several of the class I transcripts by qRT-PCR (S5 Fig). These transcripts showed a progressive reduction in BAC-Q72 mice, but remained unchanged in the Pcp2-ATXN2[Q127] line even at late time points. Of these 50, 16 genes (*Grm4*, *Igfbp5*, *Fstl5*, *Snrk*, *D8Ertd82e*, *Dusp5*, *Nab2*, *Btg1*, *Adrbk2*, *Slc25a29*, *Sty12*, *Crhr1*, *Synpr*, *Lrrtm2*, *Rit2* and *Cabp2*) were previously identified as GC-specific using translational profiling [29].

Class III transcripts were those that showed changes only in Pcp2-ATXN2[Q127] mice, but not in BAC-Q72 at an FDR>15 and a log2 ratio of change ≥|0.3|. We verified expression changes of six class III transcripts longitudinally in Pcp2-ATXN2[Q127] mice at 4, 8, and 24 weeks of age, and BAC-Q72 mice at 5, 9, 16 and 24 weeks of age, and their respective WT littermates by qRT-PCR. Five of the six transcripts showed significant and progressive reduction with age not only in Pcp2-ATXN2[Q127] mice but also in BAC-Q72 mice (S6 Fig). This is consistent with the milder behavioral phenotype seen in BAC-Q72 mice and suggests that the overlap of the transcriptomes in the two models may potentially be even greater.

### *Rgs8* transcripts are downregulated in the cerebella of BAC-Q72 mice

Changes in steady-state expression of a subset of genes preceded onset of physiological and behavioral changes in Pcp2-ATXN2[Q127] and BAC-Q72 mice. One of the most significantly down-regulated genes in both models prior to behavioral onset was *Rgs8* (regulator of G-protein signaling 8) (S1, S2, S3 Tables). RGS proteins are regulatory and structural components of G protein-coupled receptor complexes. RGS proteins (RGS7, RGS8, RGS11, RGS17 and RGSz1) are widely expressed in cerebellum and RGS8 is specifically distributed in dendrites and cell bodies of PCs [30,31]. Several reports suggest that the RGS family proteins are also associated with motor neuron functions [32,33].

The decreased steady-state level of *Rgs8* mRNA was confirmed by qRT-PCR in Pcp2-ATXN2[Q127] mice at 4, 8 and 24 weeks of age, indicating that these RNAs progressively declined with time (S7A Fig). In parallel, we also measured Rgs8 protein steady state levels in Pcp2-ATXN2[Q127] mouse cerebella at 24 weeks of age. As expected, Rgs8 protein levels were significantly reduced in Pcp2-ATXN2[Q127] mice when compared with wild-type mice (S7B Fig).

**Fig 7:**
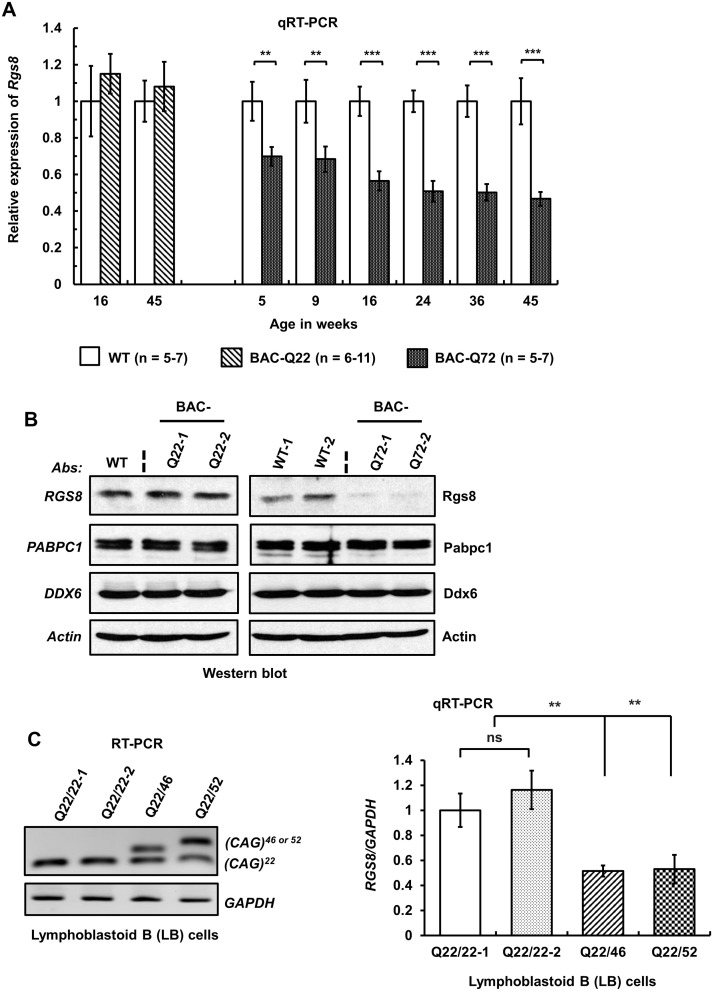
Decreased steady-state levels of *Rgs8* message and protein in BAC-Q72 mice. **(A)** qRT-PCR analyses of cerebellar RNAs from wild-type and BAC-Q22 mice show unchanged *Rgs8* levels, whereas BAC-Q72 mice show significant and progressive reduction of *Rgs8* mRNA levels starting at 5 weeks of age. n: number of animals in each group. The data are means ± SD, **p<0.01, ***p<0.001. **(B)** Western blot analyses indicate reduction of Rgs8 steady-state levels in cerebella of BAC-Q72 mice, but no change in BAC-Q22 mice when compared with wild-type mice. The blot is a representative Western blot of 3 independently performed experiments with 2 animals each per BAC line. **(C)** SCA2 patient-derived LB cells demonstrate decreased *RGS8* transcripts. Total RNAs were isolated from LB cell lines derived from two normal control individuals and two SCA2 patients and subjected to RT-PCR analysis using primers specifically amplifying the human *ATXN2* CAG repeat. RT-PCR analyses indicate the expression of ATXN2 with expanded CAG repeats (46 or 52) (left panel). qRT-PCR analyses of synthesized cDNAs from LB cells show significant reduction of *RGS8* in both SCA2-LB cell lines. The data represent mean ± SD, **p<0.01 (right panel).

Next, we investigated the fate of *Rgs8* mRNA steady-state levels in our BAC mouse models by qRT-PCR. When tested in BAC-Q72 mouse cerebella, levels of *Rgs8* mRNA progressively decreased with time but remained unchanged in BAC-Q22 mice compared with wild-type mice across all ages of mice tested (Fig 7A).

To examine whether changes in steady-state mRNA levels led to decreased protein abundance, we performed Western blot analysis to measure Rgs8 protein in wild-type and BAC transgenic mouse cerebella. Western blot analyses indicated reduced steady-state levels of Rgs8 protein in BAC-Q72 mice but not in BAC-Q22 mice when compared with wild-type mice at 24 weeks of age (Fig 7B).

To assess whether these findings replicated in human cells we analyzed EBV-transformed lymphoblastoid (LB) cells derived from a control individual and two SCA2 patients with expansions of Q46 and Q52 (Fig 7C). We could not use skin fibroblasts as this cell type does not express *RGS8*. Two SCA2-LB cells expressing Q46 or Q52 demonstrated decreased expression of *RGS8* transcript compared with control cells expressing wild-type *ATXN2* with 22 repeats. Unfortunately, LB cells do not efficiently translate *RGS8* message, so that Western blots did not allow detection of RGS8 protein in LB cells.

**Fig 8:**
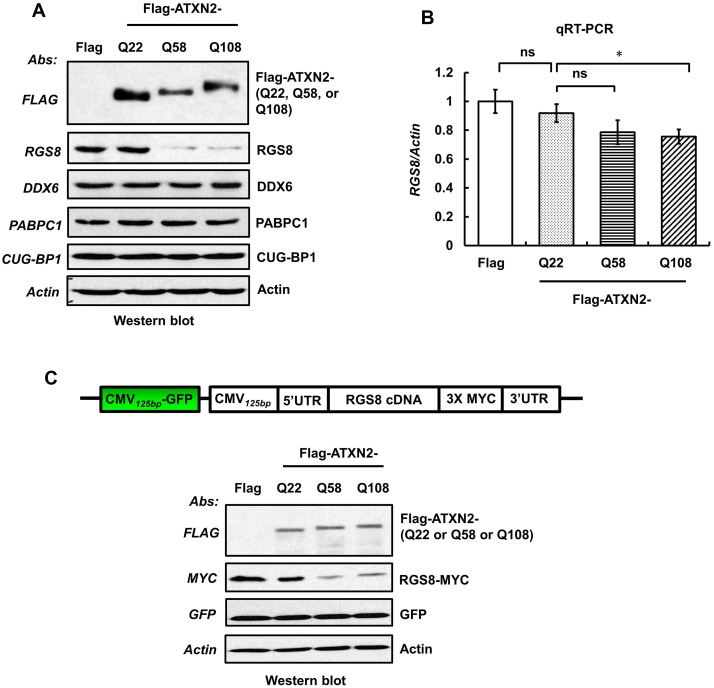
Overexpression of mutant ATXN2 in human SH-SY5Y cells recapitulates down-regulation of *in vivo* steady-state levels of Rgs8 in BAC-Q72 mice. Cells were transfected with plasmids encoding Flag-tagged cDNAs of human *ATXN2* containing Q22 or Q58 or Q108 repeats. Forty-eight hrs post-transfection, cells were selected with hygromycin (40 µg/ml) for 5–7 days and hygromycin resistant cells were harvested as two aliquots. **(A)** Protein extracts were prepared from one aliquot and subjected to Western blot analyses to measure steady-state levels of RGS8. The blots were re-probed for β-Actin as an internal loading control. **(B)** Quantitative RT-PCR analyses of synthesized cDNAs from the other aliquot demonstrate moderate reduction of *RGS8* mRNA in cells expressing Flag-ATXN2-Q108. The data are means ± SD, *p<0.05. **(C)** Mutant ATXN2 specifically induces decrease of RGS8 expression. MYC-tagged *RGS8* cDNA including 5’ and 3’ UTRs was cloned under the transcriptional control of the CMV promoter and transfected into short-term hygromycin selected SH-SY5Y cell lines expressing Flag-tagged ATXN2-Q22, -Q58 or -Q108. Forty-eight hrs post-transfection, levels of exogenous RGS8 are significantly decreased in cells expressing ATXN2-Q58 or -Q108 compared with cells expressing wild-type ATXN2-Q22. To control for equal transfection, we monitored levels of GFP, which was expressed as an independent cassette in the plasmid. Blots were re-probed for β-actin as an internal loading control. The blot represents one of three independent experiments.

To test whether reduction of Rgs8 levels induced by mutant *ATXN2* could be recapitulated *in vitro*, we measured steady-state levels of *RGS8* mRNA and protein in hygromycin selected enriched SH-SY5Y cells expressing Flag-tagged ATXN2-Q22, -Q58 or -Q108. Western blot analyses of whole cell extracts indicated that expression of ATXN2-Q58 or Q108 resulted in decreased RGS8 levels compared to control or ATXN2-Q22 (Fig 8A). To exclude that decreased RGS8 levels were a consequence of selective cellular toxicity of ATXN2-Q58 or -Q108 expression, we measured expression of endogenous DDX6 and PABPC1, which have been shown to interact with ATXN2 [6,8] and CUG-BP1, a nuclear protein by Western blot analysis. The levels of DDX6, PABPC1 and CUG-BP1 were not altered (Fig 8A) strongly supporting that the effect of mutant ATXN2 was specific to RGS8. In parallel, qRT-PCR analyses of SH-SY5Y cell lines expressing Flag-tagged wild-type and mutant ATXN2 demonstrated a moderate reduction of *RGS8* mRNA in cell expressing Flag-ATXN2-Q108 (Fig 8B).

Decrease of RGS8 levels in mutant BAC mice could be the result of transcriptional control, mRNA stability and processing or translational control. In contrast to other polyQ proteins, ATXN2 does not enter the nucleus [19] and protein interaction studies have not yielded proteins thought to be involved in transcriptional control. To examine translation of *RGS8*, we expressed exogenous RGS8 in hygromycin selected SH-SY5Y cells expressing Flag-tagged ATXN2-Q22, -Q58 or -Q108. MYC-tagged *RGS8* cDNA including 5’ and 3’ UTRs was cloned under the transcriptional control of the CMV promoter. Forty-eight hrs post-transfection, Western blot analyses revealed that the levels of exogenous RGS8 were significantly decreased in cells expressing ATXN2-Q58 or -Q108 compared with cells expressing wild-type ATXN2-Q22 (Fig 8C). To control for equal transfection, we monitored levels of GFP, which was expressed as an independent cassette in the plasmid. Thus, presence of mutant ATXN2 reduced RGS8 protein levels *in vivo* and *in vitro*.

### ATXN2 interacts with *RGS8* mRNA and regulates its expression

Reduced protein levels potentially out of proportion to reduced mRNA levels *in vivo* and *in vitro* suggested to us that ATXN2 might be directly involved in the translation or stability of specific mRNAs. In addition, ATXN2 is known to interact with RNAs through a “Like Sm (LSm) domain” [34–36]. It also interacts with cytoplasmic poly(A)-binding protein 1 (PABPC1) and assembles with polysomes [6,7]. Therefore, we first tested interaction of ATXN2 with *RGS8* mRNA and then performed *in vitro* translation assays in the presence of wild-type and mutant ATXN2.

**Fig 9:**
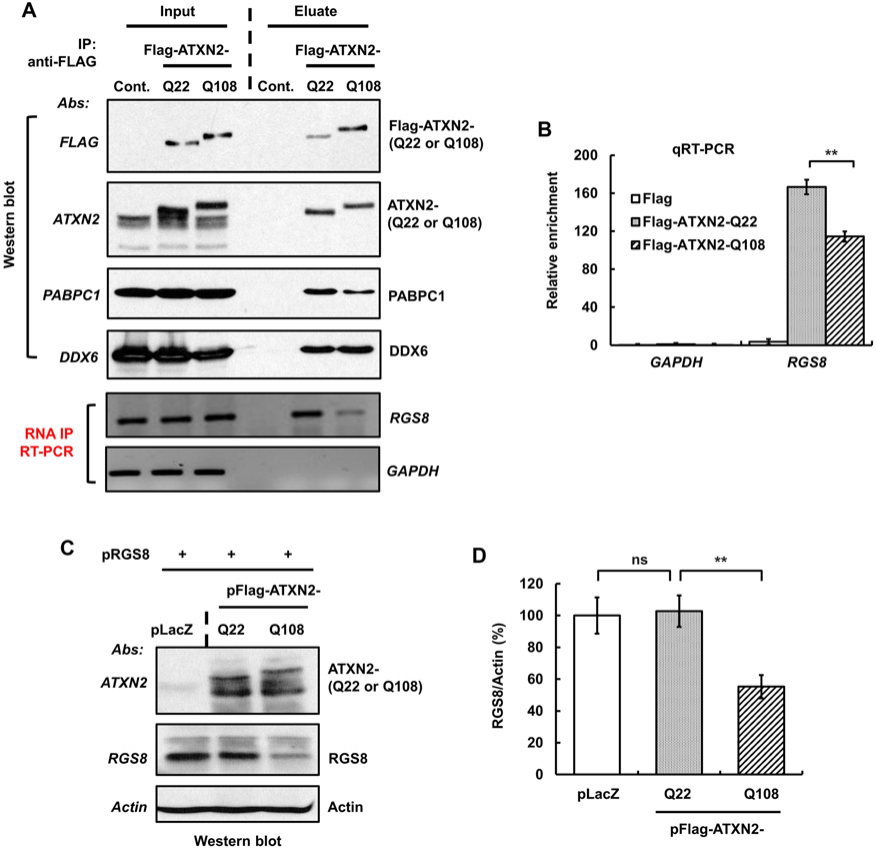
ATXN2 immunoprecipitates *RGS8* mRNA and regulates RGS8 steady state levels *in vitro*. **(A)** SH-SY5Y whole cell extracts expressing Flag-ATXN2-Q22 or Flag-ATXN2-Q108 were subjected to immunoprecipitation with a mAb to the Flag tag. After washing the beads with buffer (200 mM NaCl), bound protein-RNA complexes were eluted by Flag peptide competition. IP products were divided equally into two parts and subjected to Western blot and RT-PCR analyses to identify ATXN2 interacting proteins and RNAs. Western blot analyses of the eluted proteins show co-IP of PABPC1 and DDX6, known ATXN2 interactors. RT-PCR analyses of the second aliquot show that both Flag-ATAXN2-Q22 and Flag-ATAXN2-Q108 immunoprecipitate *RGS8* mRNA but not *GAPDH* mRNA. ATXN2-Q108 shows differential binding toward *RGS8* mRNA when compared with ATXN2-Q22. **(B)** Interaction of ATXN2 with *RGS8* mRNA determined by qRT-PCR. Synthesized cDNAs from the second aliquot of IP products (A) were subjected to qRT-PCR analyses. Interaction of *RGS8* mRNA with ATXN2-Q108 was significantly reduced when compared with ATXN2-Q22. Data are mean ± SD, n = 3 independent experiments. **p<0.01. **(C-D)** Mutant ATXN2 represses RGS8 synthesis *in vitro*. First, cDNA plasmids of LacZ (control) and Flag-tagged ATXN2-Q22 or -Q108 were added to rabbit reticulocyte lysate mixture and proteins synthesized for 2 hrs. *RGS8* cDNA plasmid was added to each translational reaction with fresh rabbit reticulocyte lysate and incubated further for 4 hrs. The synthesized RGS8 protein from each translational product was analyzed by SDS-PAGE followed by Western blot analyses. ATXN2-Q108 reduces RGS8 synthesis significantly when compared with ATXN2-Q22 (C). Quantification of RGS8 on Western blots, data are mean ± SD, n = 3 independent experiments. **p<0.01, Student’s t-test) (D). The blot represents one of three independent immunoprecipitation experiments.

We performed Protein-RNA immunoprecipitation (IP) experiments in cultured SH-SY5Y cells overexpressing Flag-tagged ATXN2 containing Q22 or Q108. Whole cell extracts were incubated with Flag-mAb-beads and immunoprecipitates were washed with a buffer containing 200 mM NaCl. Bound protein-RNA complexes were eluted from the beads by Flag peptide competition. The IP products were divided equally into two aliquots and one aliquot was analyzed by Western blot. As shown in Fig 9A, the eluted proteins showed co-IP of DDX6 and PABPC1, which are known to interact with ATXN2 [6,8]. To identify RNAs that immunoprecipitated with ATXN2, the extracted RNAs from the second aliquot were subjected to RT-PCR and qPCR analyses. Our results showed that *RGS8* mRNA precipitated with ATXN2-Q22 and ATXN2-Q108 (Fig 9A and 9B). Binding of *RGS8* mRNA with ATXN2-Q108, however, was significantly reduced compared with ATXN2-Q22 in three independent experiments.

We next proceeded to examine *in vitro RGS8* translation. For that purpose, we performed assays using Flag-tagged ATXN2 with Q22 or Q108, respectively, and determined RGS8 protein abundance by Western blot analysis. In three independent experiments, one of which is shown in Fig 9C, levels of RGS8 decreased significantly in the presence of ATXN2-Q108 when compared with the levels in the presence of ATXN2-Q22. No significant alteration in the levels of RGS8 synthesis was detected between ATXN2-Q22 and control extracts (Fig 9C and 9D). These results suggest a role for ATXN2 in translational regulation and a dysregulation of this process in the presence of mutant ATXN2.

## Discussion

We developed new BAC-SCA2 transgenic mouse lines and showed that a protein involved in G-coupled protein signaling was significantly down-regulated supporting our model of mGluR1-mediated enhanced Ca^2+^ release in SCA2. Both BAC transgenic lines express human full-length ATXN2 with Q22 or Q72 under the control of endogenous human regulatory elements. BAC-Q72, but not BAC-Q22 mice, showed motor function deficits accompanied by changes in PC morphology and steady-state mRNA levels.

Pursuing transcriptome changes in Pcp2-ATXN2[Q127] mice, we demonstrated that expression of mutant ATXN2 was associated with decreased expression of *RGS8* both *in vivo* in BAC mice and in human cell culture models. Reduced *RGS8* expression was the result of reduced interaction of *RGS8* transcript with mutant ATXN2 protein and reduced *in vitro* translation.

### Fidelity of the new BAC model

Mouse models generated with tissue specific or strong promoters facilitate the evaluation of functional and anatomical consequences in many neurological disorders. The Purkinje cell protein 2 (Pcp2) and the Prion protein (PrP) promoters have been used to generate mouse models for polyQ ataxias such as SCA1, SCA2 and SCA3 [19,20,37–41]. For instance, the use of the *Pcp2* promoter for expressing mutant *ATXN1* or *ATXN2* has been shown to recapitulate the progressive cellular and functional phenotype of human SCA1 or SCA2 [19,20,37].

Use of a BAC-transgenic approach resulted in a more widespread expression of the transgene mirroring prior observations of endogenous *ATXN2* expression in mouse and human [1]. The control regions included in our BAC transgene specified expression in CNS and non-CNS tissues (Fig 1B). In the CNS, expression was seen in the cerebral hemispheres, cerebellum and spinal cord. This is consistent with expression of endogenous mouse *Atxn2* [1] and *in situ* hybridization data as shown in the Allen Brain Atlas [28].

In the cerebellum, expression of the BAC-transgene was seen in PCs, but also in granule cells, and neurons of the dentate nucleus (Fig 2). As the transgenes were not tagged, we used LCM to establish transgene expression in these sub-regions of the cerebellum. Future physiological experiments using the cerebellar slice preparation will need to examine what role mutant *ATXN2* plays in granule cells and dentate nucleus and in overall cerebellar dysfunction in comparison with the PC-targeted expression of mutant *ATXN2* [20].

### Motor dysfunction

Motor function deficits are common to all SCA2 mouse models, although their ages of onset differ. The accelerating rotarod is used to measure motor coordination and motor learning over a number of days. Our BAC-Q72 mice developed progressive motor deficits beginning at 16 weeks of age (Fig 3B). The motor phenotype of our BAC-Q72 mice was intermediate to that of our Pcp2-ATXN2[Q58] and Pcp2-ATXN2[Q127] mice, although transgene copy numbers and precise developmental expression patterns are difficult to compare.

As with our Pcp2-ATXN2[Q22] line [19], the BAC-Q22 line did not show a motor or cellular phenotype. This study now extends these observations to mRNA measurements of key PC genes out to 45 weeks of age (Fig 5A). Lack of mRNA changes in BAC-Q22 are likely not due to differences in expression levels between lines, as transgenic *ATXN2* had higher expression in the BAC-Q22 than in the BAC-Q72 line, both at the level of mRNA and protein (Fig 1C and 1D). Lack of any changes in genes that are typically altered early in Pcp2-ATXN2[Q127] and BAC-Q72 supports the notion that simple overexpression of human wild-type *ATXN2* does not cause significant PC pathology.

In contrast, motor function deficits in *Atxn2*-CAG42 knock-in mice were not evident until the age of 18 months [21]. By comparing the motor functions in these four SCA2 transgenic mouse models, it is apparent that motor function deficits are dependent on CAG repeat length. Consistent with this interpretation, knock-in *Atxn1*-CAG78 SCA1 mice developed neither ataxic behavior nor a neuropathological phenotype [42], while knock-in *Atxn1*-CAG154 SCA1 mice did [43]. Our BAC-Q72 transgenic mouse model, although generating lower levels of mutant ATXN2 expression in the cerebellum, develop motor deficits that resemble findings in human SCA2 patients. These observations validate the notion that SCAs can be accurately modeled in mice.

### Body weight

Animal models for several polyQ diseases have shown alteration of body weight [21,43–45]. In this study, BAC transgenic mice demonstrated reduced body weights. The magnitude was similar to knock-in *Atxn2*-CAG42 mice and *Atxn1-*Q154/2Q mouse models [21,43]. On the other hand, mice lacking *Atxn2* exhibit obesity as a consequence of insulin resistance and altered lipid metabolism pathways [16,17,46]. Increased weight loss due to reduced body fat has also been reported in other polyglutamine diseases, including Huntington disease [47,48]. Of note, reductions in body weight were similar for BAC-Q22 and BAC-Q72 mice suggesting that with regard to the body weight phenotype a simple gain of function may be operative that is mirrored by obesity in loss of function models.

### PC morphology & mRNA levels of PC-expressed genes

#### Morphology

Purkinje cells abnormalities similar to our Pcp2-ATXN2[Q127] mice were observed in the mutant BAC mice. Abnormal PC morphology with shrunken dendritic trees, and concomitant reduction of *Calb1* and *Pcp2* steady-state levels were seen in BAC-Q72 mice at 24 weeks of age (Fig 4), but were not seen at 12 weeks of age (S4B and S4C Fig). Similarly, degeneration of cerebellar PCs has also been described as a feature of animal models of neurodegenerative diseases, including SCA1, SCA3, SCA7 and SCA17 [23,37,38,49].

#### Transcriptome & expression of PC-specific genes

We had examined expression levels of key PC genes in our Pcp2-ATXN2[Q127] mice and found that some genes showed decreased expression even prior to or at symptom onset (*Calb1*, *Pcp2*), whereas others changed relatively late (*Itpr1*). This pattern was essentially conserved in the BAC-Q72 line (Fig 5B). No expression changes were detected in the BAC-Q22 line (Fig 5A).

In order to obtain an unbiased assessment of transcriptome changes we used deep RNA-sequencing of cerebella at 8 weeks of age, thus prior to behavioral or morphological changes in BAC-Q72 mice. A large number of transcripts were changed in BAC-Q72 mice (Fig 6) in contrast to the paucity of changes observed in the Atxn2-CAG42 knock-in mice even at 18 months of age and in the homozygous state [21].

Transcriptome analysis indicated a set of mRNA changes shared with the Pcp2-ATXN2[Q127] model, but also a distinct set of genes only changed in the BAC-Q72 model (Fig 6 and S1, S2, S3 Tables). Many of the most changed transcripts in BAC-Q72 mice at 8 weeks of age were in fact primarily expressed in GCs, for example *Dusp5*, *Mybpc3*, *Snrk* and *Neurod1* (S5 Fig). This is consistent with expression of the BAC transgene in GCs as shown by LCM (Fig 2E) and indicates that GC pathology may contribute to the motor phenotype in BAC-Q72 mice.

Expression profiling studies using microarray technology of many polyQ diseases have shown transcriptome changes in cerebellar genes [50–55]. Although it is difficult to compare across different models, time points and technologies, expression changes common to these models can be found, including *Pcp2*, *Pcp4* and *Calb1*. This may point to common final pathways in autosomal dominant polyQ diseases. Of note, these genes were not changed in the Atxn2-CAG42 knock-in model [21].

Our results further support the notion that expression changes precede motor dysfunction as now shown in two independent SCA2 models. Expression changes closely mirrored progression of motor dysfunction in the BAC-Q72 line, but were absent in BAC-Q22 mice.

### RGS protein function

RGS proteins comprise a large family of more than 20 members that negatively modulate heterotrimeric G protein signaling. They share a homologous RGS domain that functions to activate the GTPase of Gα proteins. RGS8 is widely expressed in testis, brain, and cerebellar Purkinje cells [56,57]. Mice lacking *Rgs6* or *Rgs9* exhibit motor function deficits and ataxia [32,33]. *Rgs8* knock-out mice were viable, fertile, and showed normal development, but have not been tested in detail for motor behaviors or PC morphology [57].

### ATXN2 and RNA metabolism

Given the importance of a dysregulated mGluR1-ITPR1 axis in SCA2 pathology [13,58], reduction in RGS proteins could further increase abnormally enhanced mGluR1 signaling. We therefore examined RGS8 abundance in BAC-Q72 mice and Epstein-Barr virus immortalized human lymphoblastoid B (LB)-cells from SCA2 patients (Fig 7). The results demonstrated that *Rgs8* transcripts and protein abundance were significantly decreased in BAC-Q72 mice (Fig 7A and 7B). Consistent with this, SCA2-LB cells also demonstrated decreased *RGS8* transcripts (Fig 7C). Next, we developed an *in vitro* model using SH-SY5Y cells. Overexpression of mutant ATXN2 resulted in downregulation of RGS8 and this phenomenon was not seen for other known ATXN2 interactors (Fig 8).

As protein levels appeared somewhat depressed out of proportion to the observed reduction in steady-state mRNA levels, we hypothesized that ATXN2 might regulate translation of mRNAs directly. Consistent with this hypothesis, we showed that both wild-type and mutant ATXN2 immunoprecipitated *RGS8* mRNA in human cell culture and that this interaction was weaker for mutant ATXN2 (Fig 9A and 9B). This was also reflected in i*n vitro* translation assays as presence of an expanded polyQ tract in ATXN2 reduced translation (Fig 9C and 9D).

Our observations are consistent with studies of the *Drosophila* homolog of *ATXN2* (*Atx2*). Atx2 regulates PERIOD (PER) translation by interacting with TWENTY-FOUR (TYF) that is required for circadian locomotor behavior. Depletion of *Atx2* or expression of mutant Atx2 protein blocked the recruitment of PABP to the TYF-containing protein complex and decreased abundance of PER, thereby altering behavioral rhythms [59,60]. ATXN2 interactions with polyA-binding protein 1 (PABPC1), the splicing factor A2BP1/FOX1 and poly-ribosomes further support roles for ATXN2 in RNA metabolism [5–7]. Depletion of PABP from a cell free extract prevented initiation of mRNA translation [61]. Our studies now extend these observations to mammalian systems and to a gene abundantly expressed in PCs. It is quite likely that *Rgs8* will be just one member of a larger set of mRNAs whose expression is regulated by ATXN2.

### mRNA metabolism

Aberrant RNA metabolism including processing, degradation, and translation is now recognized to play an important role in neurodegenerative diseases. Among these diseases are amyotrophic lateral sclerosis (ALS), Spinal Muscular Atrophy (SMA) and Fragile X syndrome (FXS) [62–70]. Although ATXN2 had been implicated in steps regulating mRNA translation and formation of stress granules [8,71,72], to our knowledge we describe for the first time a significant difference in these functions between wild-type and mutant ATXN2. Our observations may also have implications for ALS as long normal *ATXN2* alleles are a risk factor for ALS [18,73] and some individuals with full mutant *ATXN2* alleles may present as ALS [74].

In summary, BAC-SCA2 transgenic mice represent the first animal model with expression of mutant full-length human *ATXN2* under the control of its endogenous human promoter including intronic regulatory sequences. These sequences resulted in widespread expression of *ATXN2* mirroring expression of endogenous *Atxn2*. Expression of mutant ATXN2-Q72, but not wild-type ATXN2-Q22, led to a progressive motor deficit, accompanied by morphological and transcriptome changes. As previously demonstrated in *C*. *elegans* and the fly [6, 59,60,75], *ATXN2* may exert translational control upon a subset of mRNAs. We showed in two independently generated models that presence of mutant ATXN2 *in vivo* resulted in reduced steady-state levels of *RGS8* mRNA and even further reduction in RGS8 protein. ATXN2 coprecipitated with *RGS8* mRNA and mutant ATXN2 reduced translation of *RGS8* mRNA. RGS proteins can act via Gα_q_ on G-protein coupled receptors. As mutant ATXN2 enhances Ca^2+^ release from the endoplasmic reticulum (ER) via its abnormal interaction with ITPR1, reduction of RGS8 might be predicted to further increase intracellular Ca^2+^ by prolonging mGluR1 stimulated Ca^2+^ release. Our studies now provide a framework to further examine the aberrant mGluR1-ITPR1 axis in SCA2 pathogenesis.

## Materials and Methods

### Ethics statement

Human lymphoblastoid B (LB)-cells from SCA2 patients and unaffected normal controls were used. All subjects gave written consent and all work was approved by the Institutional Review Board at the University of Utah under IRB# 00035351 and IACUC- University of Utah IACUC committee, protocol number 13–0004. BAC-SCA2 mice were maintained in FVB background and bred and maintained under standard conditions consistent with National Institutes of Health guidelines and approved by the University of Utah, IACUC protocol.

### Generation of BAC-SCA2 transgenic mice

A 169 kb of RP11-798L5 BAC clone (Empire Genomics., USA) containing the 150 kb human *ATXN2* locus was engineered to replace the endogenous *ATXN2* exon-1 CAG22 with CAG72 repeats. The BAC DNA was prepared according to published protocols [76,77] and microinjected into FVB fertilized eggs to produce transgenic mice at the University of California Irvine (UCI) Mouse Core Facility. BAC-SCA2 mice were maintained in the FVB background and bred and maintained under standard conditions consistent with National Institutes of Health guidelines and approved by the University of Utah, IACUC protocol. For genotyping of BAC-SCA2 transgenic mice, DNA was isolated from mice tails using Qiagen genomic DNA extraction kit (Qiagen Inc., USA) and genotyping PCR was performed. Three primer sets were used to identify the transgene and the primer sequences are follows: P3 forward: 5’-AATTTATGTGATGTT CACTGTTTCTTCC-3’, P3 reverse: 5’-TACGGTCCCTCCAAATAGTGTTAC-3’, P7 forward: 5’-TCTTTTTACAGTACAAGCCCACCACC-3’, P7 reverse: 5’-TTCAAAATG CACCCTTAGCACACCTG-3’, SCA2-A forward: 5’-GGGCCCCTCACCATGTCG-3’, SCA2-B reverse: 5’-CGGGCTTGCGGACATTGG-3’. For all experiments wild-type and transgenic animals were kept as littermates. From 3 to 5 litters were used per experiment dependent on actual size of litters.

### RNA expression analyses by RT-PCR and quantitative RT-PCR

Mice were deeply anesthetized with isoflurane. Mouse cerebella were removed and immediately submerged in liquid nitrogen. Tissues were kept at −80°C until the time of processing. Total RNA was extracted from mouse cerebella using the RNeasy Mini Kit (Qiagen Inc., USA) according to the manufacturer’s protocol. DNAse I treated RNAs were used to synthesize cDNAs using the ProtoScript cDNA First Strand cDNA Synthesis Kit (New England Biolabs Inc., USA). Primers for RT-PCR were designed to prevent amplification from genomic DNA (annealing sites in different exons or across intron-exon boundaries). Human *ATXN2* primer sites were in exon 1 and exon 5, including Exon 1-F (5’-CTCCTCGGTGGTCGCGGCGACCTC-3’) and Exon 5-R (5’-CTCTTTTTGCATAACT GGAGTCC-3’). *ATXN2* primers for amplifying CAG repeats were SCA2-A (5’-GGGCCCCTCACCATGTCG-3’) and SCA2-B (5’-CGGGCTTGCGGACATTGG-3’). *Gapdh* primers were GAPDH-F (5’-TGAAGGTCGGA GTCAACGGATTTGG-3’ and GAPDH-R (5’-GGAGGCCATGTGGGCCATGAG-3’). *Gapdh* amplification was conducted in parallel as an internal control for RNA quality and was also employed to evaluate quality the reverse transcriptase reactions. Quantitative RT-PCR was performed in Bio-Rad CFX96 (Bio-Rad Inc., USA) with the Power SYBR Green PCR Master Mix (Applied Biosystems Inc, USA). PCR reaction mixtures contained SYBR Green PCR Master Mix and 0.5 pmol primers and PCR amplification was carried out for 45 cycles: denaturation at 95°C for 10 sec, annealing at 60°C for 10 sec and extension at 72°C for 40 sec. The threshold cycle for each sample was chosen from the linear range and converted to a starting quantity by interpolation from a standard curve run on the same plate for each set of primers. All gene expression levels were normalized to the *Actin* or *Gapdh* mRNA levels. Primer pairs designed for qRT-PCR are given as forward and reverse, respectively, and listed in supplementary table (S5 Table).

### RNA sequence

Cerebella from 8 weeks old wild-type and BAC-Q72 mice (4 animals in each group), and 6 weeks old Pcp2-ATXN2[Q127] and wild-type littermates (16 animals in each group) were used for RNA sequence analyses. Total RNA was isolated using miRNeasy Mini Kit (Qiagen Inc., USA) according to the manufacturer’s protocol. RNA quality was determined using the Bioanalyzer 2100 Pico Chip (Agilent). Samples with an RNA integrity number (RIN) >8 were used for library preparation using Illumina TrueSeq Stranded Total RNA Sample Prep with Ribo-Zero rRNA Removal Kit for mouse. Single-end 50-bp reads were generated on a Hiseq 2000 sequencing machine at the University of Utah Microarray and Genomic Analysis Shared Resource using Illumina Version 4 flow cells. Reads were then aligned to the mouse reference genome (mm10) by Novoalign (http://www.novocraft.com). Quality of RNA sequencing was extremely high with an average of twenty eight million reads for BAC-Q72 and twenty two million reads for Pcp2-ATXN2[Q127]. Ninety eight percent of the reads for both sets of RNAs were aligned to the reference mouse genome. After read alignment, differentially expressed genes were identified using the DRDS application (version 1.3.0) in the USeq software package (http://useq.sourceforge.net/). Gene Ontology (GO) annotations were obtained for all differentially expressed genes (p<0.05). GO enrichment results were obtained using the software DAVID [78,79]. Overlap of BAC-Q72 and Pcp2-ATXN2[Q127] molecular function GO annotations was accomplished using only level 5 categories (p<0.05).

### Cell culture

SH-SY5Y cells were cultured and maintained in DMEM media containing 10% fetal bovine serum. Epstein-Barr virus immortalized human lymphoblastoid B (LB)-cells from SCA2 patients and unaffected normal controls were cultured in RPMI 1640 medium supplemented with 15% fetal bovine serum, penicillin and streptomycin. All subjects gave written consent and all work was approved by the Institutional Review Board at the University of Utah.

### Preparation of protein lysates and western blot analyses

Protein extracts were prepared by homogenization of mouse cerebella in extraction buffer (25 mM Tris-HCl pH 7.6, 300 mM NaCl, 0.5% Nonidet P-40, 2 mM EDTA, 2 mM MgCl_2_, 0.5 M urea and protease inhibitors; Sigma; cat# P-8340) followed by centrifugation at 4°C for 20 min at 16,100 × *g*. Only supernatants were used for Western blotting. Cellular extracts were prepared by the single-step lyses method [80]. The cells were harvested and suspended in SDS-PAGE sample buffer (2x Laemmli Sample Buffer; Bio-Rad; cat# 161–0737) and then boiled for 5 min. Equal amounts of the extracts were subjected to Western blot analysis to determine the steady-state levels of proteins using the antibodies listed below. Protein extracts were resolved by SDS-PAGE and transferred to Hybond P membranes (Amersham Bioscience Inc., USA). After blocking with 5% skim milk in 0.1% Tween 20/PBS, the membranes were incubated with primary antibodies in 5% skim milk in 0.1% Tween 20/PBS for 2 hrs at room temperature or overnight at 4°C. After several washes with 0.1% Tween 20/PBS, the membranes were incubated with the corresponding secondary antibodies conjugated with HRP in 5% skim milk in 0.1% Tween 20/PBS for 2 hrs at room temperature. Following three additional washes with 0.1% Tween 20/PBS, signals were detected by using the Immobilon Western Chemiluminescent HRP Substrate (Millipore Inc., USA; cat# WBKLSO100) according to the manufacturer’s protocol. The following antibodies were used throughout the study. ATXN2 mAb [(1:3000), BD Biosciences Inc.; cat# 611378], 5TF1-1C2 mAb [(1:3000), Millipore Inc.; #MAB1574], RGS8 rabbit polyclonal Ab [(1:3000), Novus Biologicals; #NBP2-20153], Calbindin-D-28K mAb [(1: 5000), Sigma Inc.; cat# C9848], PCP2 mAb [(1: 5000), Santa Cruz Inc.; cat# sc-137064], DDX6 rabbit polyclonal Abs [(1:4000), Santa Cruz Inc.; cat# sc-27127-R], PABPC1 mAb [(1:4000), Santa Cruz Inc.; cat# sc-27127-R], CUG-BP1 mAb [(1:4000), Santa Cruz Inc.; cat# sc-20003], Flag M2 mAb [(1:10,000), Sigma Inc.; cat# F3165], GFP mAb [(1:3000), Santa Cruz Inc.; cat# sc-9996] and MYC mAb conjugated with HRP [(1:5000), Invitrogen Inc.; cat# A3858]. To control for protein quality and loading, the membranes were re-probed with β-Actin mAb conjugated with HRP [(1:10,000), Sigma Inc.; cat# A3858]. The secondary antibodies were goat anti-mouse IgG-HRP [(1:5000), Sigma Inc.; cat# A2304], and donkey anti-rabbit IgG-HRP [(1:5000), Santa Cruz Inc.; cat# sc-2057].

### Behavioral analyses

Motor behavior of SCA2 mice was determined using the accelerating rotarod. Cohorts were age matched prior to all behavioral experiments. Male and female mice performed equally well; therefore, data were pooled and gender differences were not evaluated further. The motor performance of BAC-Q22 and BAC-Q72 mice and wild-type littermates were evaluated using the accelerating rotarod (Ugo Basile) according to our published protocol [20]. For mice clinging to the rod, the time at which a mouse had completed 5 rotations was taken as the final latency.

### Immunohistochemistry

Mice were deeply anesthetized with isoflurane, then transcardially perfused with ice-cold phosphate buffered saline (PBS). Tissue was quickly removed and submerged into cold 4% paraformaldehyde (Electron Microscopy Sciences) and kept at 4°C overnight. The following day, PFA was replaced with 10 mM sodium citrate pH 6.0, and then incubated at 4°C overnight, after which the tissue was exposed to microwave radiation 3 times in 10 sec bursts. Following microwave radiation, tissues were cryoprotected by incubating in 20% sucrose in PBS overnight followed by 30% sucrose overnight both at 4°C. Then the samples were mounted in Tissue-Tek O.C.T. Compound (Sakura Finetek) and stored at -80°C until the time of sectioning. Tissue sections were cut into 20 μM thick slices and floated into cold PBS. Tissues were washed 3 times with PBS at RT for 15 min each time. Free-floating sections were incubated with blocking/permeabilization solution consisting of 5% skim milk, 0.3% Triton X-100 in PBS for 4 hr at RT. Sections were then incubated overnight at 4°C with primary antibodies, calbindin-28kDa mAb at 1:200 dilution. After 3 washes in PBS at 15 min each, sections were incubated with DyLight-550 (Red) (Thermo Fischer Scientific) fluorescent secondary antibodies at 1:500 dilution for 2 hr at RT. Following incubation, the sections were washed 3 times with PBS, and the sections were transferred to Superfrost Plus microscope slides (Fischer Scientific) and mounted with Prolong Gold (Invitrogen). Sections were imaged using confocal microscope (Nikon Eclipse Ti microscopy) and analyzed by Nikon EZ-C1 software. PCs were counted in parasagittal slices from 3 mice in each group.

### Laser capture microdissection (LCM)

Fresh whole cerebella from wild type or BAC-Q22 or BAC-Q72 mice was freeze-mounted in O.C.T. and sectioned onto Arcturus PEN Membrane glass slides. Sections were fixed and H&E stained using the Fast Frozen Stain Kit (EMS). Sections on slides were then dehydrated by passage through a solution series of 95% ethanol, 100% ethanol, and then xylene. Prepared slides were stored in a desiccated chamber until needed. LCM was performed using the Arcturus Veritas LCM system. RNAs were prepared from tissue on LCM caps (CapSure, Applied Biosystems) using the Arcturus PicoPure RNA Kit (Applied Biosystems Inc., USA). RNA yield was typically 5 μg/cap. cDNA was then prepared by using the ProtoScript M-MuLV First Strand cDNA Synthesis Kit (NEB Inc., USA) and used for qRT-PCR as described in Methods above.

### Immunoprecipitations

To identify proteins and RNAs that bind to ATXN2, we carried out protein-RNA immunoprecipitation (IP) experiments from lysates of SH-SY5Y cells expressing Flag-ATXN2-Q22 and Flag-ATXN2-Q108. Whole cell extracts were prepared by the two-step lyses method [80]. First, cells were lysed with a cytoplasmic extraction buffer (25 mM Tris-HCl pH 7.6, 10 mM NaCl, 0.5% NP40, 2 mM EDTA, 2 mM MgCl_2_, protease and RNAse inhibitors) and cytoplasmic extracts were separated by centrifugation at 14,000 RPM for 20 min. Second, the resultant pellets were suspended in nuclear lysis buffer or high salt lyses buffer (25 mM Tris-HCl, pH 7.6, 500 mM NaCl, 0.5% Nonidet P-40, 2 mM EDTA, 2 mM MgCl_2_, protease and RNAse inhibitors), and the nuclear extracts were separated by centrifugation at 14,000 RPM for 20 min. The nuclear extracts were then combined with the cytoplasmic extracts and denoted as whole cell extracts. Specifically, while combining cytoplasmic and nuclear extracts, the NaCl concentration was adjusted to physiologic buffer conditions (~150 mM) to preserve *in vivo* interactions. Ninety percent of cell extracts were subjected to Flag monoclonal antibody (mAb) IP (Anti-Flag M2 Affinity Gel, Sigma Inc.; cat# A2220-1ML) to immunoprecipitate ATXN2 interacting protein-RNA complexes. The remaining 10% of whole cell extracts were saved as the input control for Western blotting and RT-PCR analyses. The IPs were washed with a buffer containing 200 mM NaCl and the bound protein-RNA complexes were eluted from the beads with Flag peptide competition (100 μg/ml). Eluted fractions were divided into two equal parts. One part was analyzed by SDS-PAGE followed by Western blotting to determine the efficiency and quality of immunoprecipitation. RNA was isolated from the other fraction and subjected to RT-PCR and qRT-PCR analyses to identify RNAs that bound to wild type or mutant ATXN2.

### *In vitro* translation assay

To determine the role of ATXN2 on *RGS8* mRNA translation, *in vitro* translation assays were performed using the rabbit reticulocyte lysate-based cell free TNT Quick Coupled Transcription/Translation Kit (Promega Inc., USA) according to the manufacturer’s instructions, with minor modifications. Briefly, 1 μg of cDNA plasmids of LacZ (control) and Flag-tagged *ATXN2* expressing Q22 or Q108 were added to 20 μl of the rabbit reticulocyte lysate kit component, including 20 μM amino acids in a total volume of 25 μl. The translation reaction was carried out for 2 hr at 30°C. Next 1 μg of *RGS8* cDNA plasmid was added to each translation reaction with fresh rabbit reticulocyte lysate containing 20 μM amino acids in a total volume of 50 μl, and incubated further at 30°C for 4 hr. Translation assays was analyzed by SDS-PAGE followed by Western blot analyses.

### Statistical analysis

For Western blot analyses, the experiments were performed at least three times, and wherever appropriate gel films were scanned and band intensities were quantified by ImageJ analyses. The p values were calculated by pairwise Student’s t-tests. Student’s t-tests were also used to compare mRNA steady state levels between BAC and wild-type mice determined by qRT-PCR. The level of significance was set at p<0.05. In the figures, a single asterisk indicates p<0.05, a double asterisk p<0.01, a triple asterisk p<0.001, and ns represents p≥0.05. For accelerating rotarod analyses, repeated measures ANOVA was used with post-hoc t-tests to compare means.

## Supporting Information

S1 Fig Construction of BAC-SCA2 transgenic vectors and their characterization. **(A)** Schematic representation of the modified 169 kb BAC containing the entire human 150 kb *ATXN2* locus, plus 16 kb 5’ flanking and 3 kb 3’ flanking region. The BAC was engineered to replace the endogenous *ATXN2* exon-1 [(CAG)^22^ or Q22] with mutant *ATXN2* exon-1 with (CAG)^72^ encoding 72 polyQ repeats. **(B)** Southern blot analysis of BAC-SCA2 constructs is shown. The BAC DNAs were digested with *Sac*II and subjected to Southern blot analysis using the probe indicated. The results show bands with the correct sizes in the modified BAC (BAC-Q72). **(C)** Restriction enzyme mapping on a pulse-field gel electrophoresis was used to generate DNA fingerprints of BAC-Q22 (baseline unmodified human *ATXN2* BAC) and BAC-Q72. The BAC DNAs were digested with four rare cutting restriction enzymes; *Pme*I, *Sfi*l, *Not*I and *Age*I. This analysis did not reveal any new or missing fragments in BAC-Q72 vector compared to the BAC-Q22, indicating absence of rearrangement or deletions in the modified BACs.
(TIF)

S2 Fig Expression of *ATXN2* overlapping genes is not altered in BAC-Q72 mice. **(A)** Genomic organization of the human *ATXN2* region and intervening genes. **(B-D)** Relative expression levels of each intervening gene are assessed by qRT-PCR in wild-type, BAC-Q22, and BAC-Q72 cerebella with respect to actin or human *ATXN2*. *U7*.*1–202* snRNA expression levels do not change significantly between BAC-Q22 and BAC-Q72 animals when normalized to either *actin* or human *ATXN2* (B). Relative expression levels of *RP11-686G8*.*1–001* and *RP11-686G8*.*2–001* are comparable in BAC-Q22 and BAC-Q72 animals when normalized to *actin* (C, D; left panels). However normalization with human *ATXN2* does not result in significant differences between BAC-Q22 and BAC-Q72 animals (C, D; right panels). Three animals from each group were used for these analyses. Data are means ± SD, **p<0.01, Student t-test.
(TIF)

S3 Fig Regional expression of the human *ATXN2* transgene in brain and spinal cord in BAC-Q72 mice. Expression of h*ATXN2* and mouse *Atxn2* mRNAs in different regions of BAC-Q72 and wild-type mouse brains are shown. Quantitative RT-PCR was used to determine transcript levels from RNA isolated from brain sub-regions as indicated in the graph. Three animals per group were used for these analyses. The error bars indicate ± SD.
(TIF)

S4 Fig (A) Motor phenotype of ATXN2 BAC transgenic mice on the accelerating rotarod (see also Fig 3). Rotarod performance did not differ significantly in BAC-Q72 mice at 12 weeks of age compared with wild-type littermates in contrast to significantly poorer performance at 24 weeks of age. Data represent the mean ± SEM of three trials on the test day (day 3). Number of animals tested are shown within the bars. Significance was determined using repeated measures ANOVA with post-hoc test correction. ***p<0.001. **(B)** No morphological changes in the cerebellum of BAC-Q72 mice at 12 weeks of age. Representative micrographs of calbindin-28k immunostaining of PCs in the cerebellum of BAC-Q72 and wild-type littermate are shown. **(C)** PC counts did not differ significantly in BAC-Q72 mice at 12 weeks of age compared with wild-type littermates. Three animals from each group were used. The data are mean ± SD.
(TIF)

S5 Fig Validation of transcript changes (class I) of BAC-Q72 mice by qRT-PCR (see Fig 6). Cerebellar RNAs from BAC-Q72 and Pcp2-ATXN2[Q127] mice, and their respective WT littermates at different ages were used. Genes tested are; Dual specificity phosphatase 5 (*Dusp5)*, Myosin binding protein C, cardiac *(Mybpc3)*, SNF related kinase (*Snrk*) and Neurogenic differentiation 1 (*Neurod1*). BAC-Q72 mice show significant and progressive reductions of all transcripts with age when compared with their respective WT littermates. In contrast, all transcripts remained unchanged throughout in Pcp2-ATXN2[Q127] mice compared with their respective WT littermates. Gene expression levels were normalized to beta-actin. n: animal numbers for each genotype and age group are listed in brackets. Data are means ± SD, *p<0.05 **p<0.01, ***p<0.001, Student t-test.
(TIF)

S6 Fig qRT-PCR validation of a subset of class III transcripts. All of the tested transcripts show significant and progressive reduction with age not only in Pcp2-ATXN2[Q127] mice as expected by RNA-seq, but also in BAC-Q72 mice. Only *Cacna1g* showed late reduction at 24 weeks. Cerebellar RNAs from Pcp2-ATXN2[Q127] and BAC-Q72 mice, and their respective WT littermates were analyzed at the indicated time points. Genes tested are; *Grid2*, Tubulin tyrosine ligase-like family, member 5 (*Ttll5)*, Phosphodiesterase 5A, cGMP-specific (*Pde5a)*, Calcium channel, voltage-dependent, T type, alpha 1G subunit (*Cacna1g)*, Inositol polyphosphate-4-phosphatase, type I *(Inpp4a)* and Potassium large conductance calcium-activated channel, subfamily M, alpha member 1 (*Kcnma1)*. Gene expression levels were normalized to beta-actin. n: animal numbers for each genotype and age group are listed in brackets. Data are means ± SD, *p<0.05 **p<0.01, ***p<0.001, Student t-test.
(TIF)

S7 Fig Decreased steady-state levels of Rgs8 message and protein in Pcp2-ATXN2[Q127] transgenic mice. **(A)** qRT-PCR analyses of cerebellar RNA from wild-type and Pcp2-ATXN2[Q127] mice show significant and progressive reduction of *Rgs8* mRNA levels. n: number of animals in each group. The data are means ± SD, ***p<0.001, Student’s t-test. **(B)** Western blot analyses indicate reduction of Rgs8 steady-state levels in Pcp2-ATXN2 [Q127] mouse cerebella when compared with wild-type mice at 24 weeks of age. Three wild-type and three transgenic animals were tested. The depicted blot is representative of one of 3 independent Western blot experiments.
(TIF)

S1 Table Top 50 (by FDR) differentially expressed transcripts in BAC-Q72 mice. (XLSX)

S2 Table Top 50 (by FDR) differentially expressed transcripts in Pcp2-ATXN2[Q127] mice. The respective changes in BAC-Q72 mice are listed on the right side of the table for comparison.
(XLSX)

S3 Table Top 50 (by FDR in BAC-Q72 mice) differentially expressed shared transcripts (class II) between BAC-Q72 and Pcp2-ATXN2[Q127] mice. (XLSX)

S4 Table Gene Ontology (GO) analysis of differentially expressed transcripts in BAC-Q72 and Pcp2-ATXN2[Q127] mice. (XLSX)

S5 Table qRT-PCR primer sequences. (XLSX)
