# Supplementary figures and images for: Ataxin-2 Regulates RGS8 Translation in a New BAC-SCA2 Transgenic Mouse Model
Source: PLoS Genet. 2015 Apr 22;11(4):e1005182. doi: 10.1371/journal.pgen.1005182 (PMC4406435; doi:10.1371/journal.pgen.1005182)

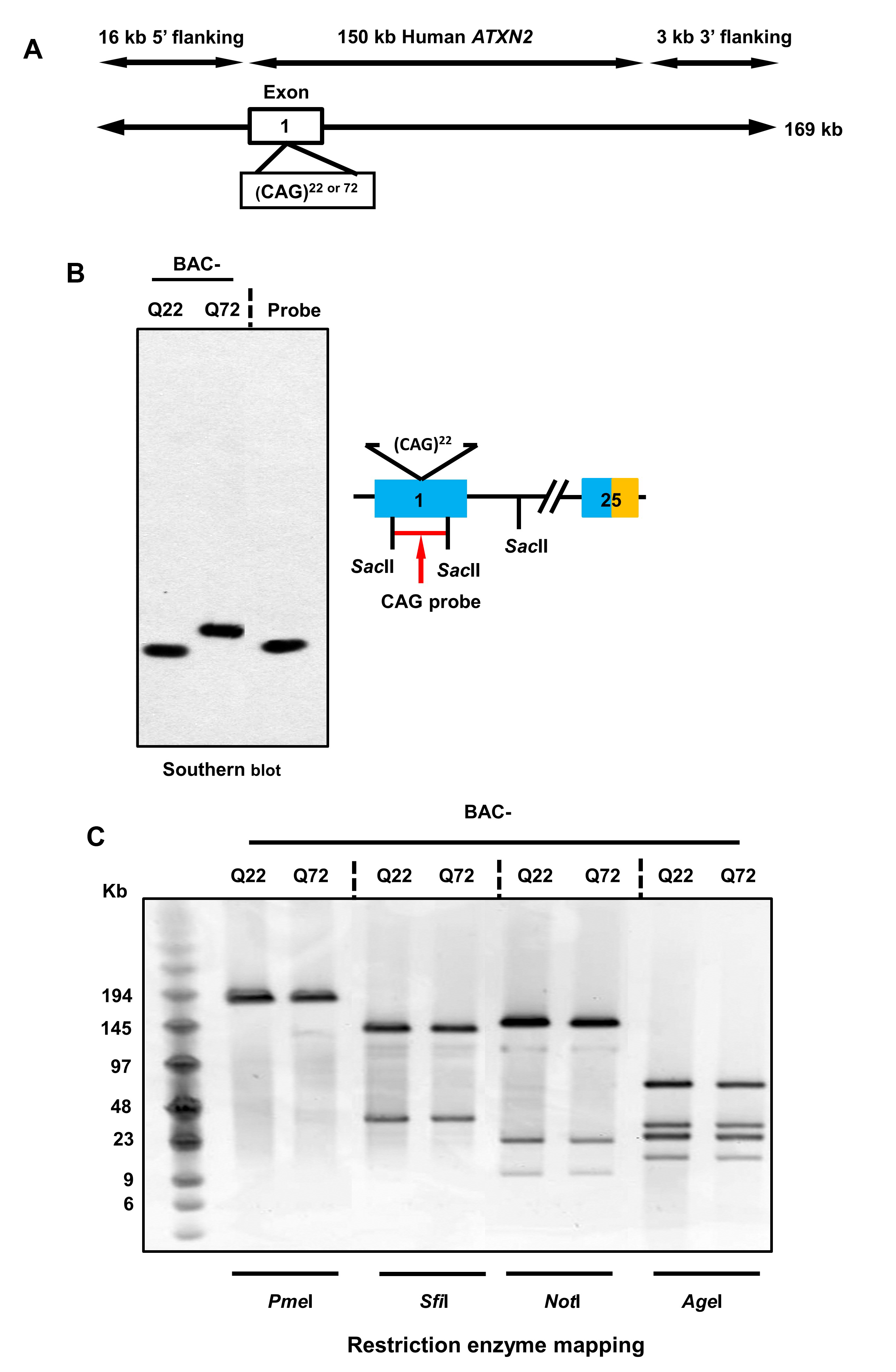

Supplement: S1 Fig — (A) Schematic representation of the modified 169 kb BAC containing the entire human 150 kb ATXN2 locus, plus 16 kb 5’ flanking and 3 kb 3’ flanking region. The BAC was engineered to replace the endogenous ATXN2 exon-1 [(CAG)22 or Q22] with mutant ATXN2 exon-1 with (CAG)72 encoding 72 polyQ repeats. (B) Southern blot analysis of BAC-SCA2 constructs is shown. The BAC DNAs were digested with SacII and subjected to Southern blot analysis using the probe indicated. The results show bands with the correct sizes in the modified BAC (BAC-Q72). (C) Restriction enzyme mapping on a pulse-field gel electrophoresis was used to generate DNA fingerprints of BAC-Q22 (baseline unmodified human ATXN2 BAC) and BAC-Q72. The BAC DNAs were digested with four rare cutting restriction enzymes; PmeI, Sfil, NotI and AgeI. This analysis did not reveal any new or missing fragments in BAC-Q72 vector compared to the BAC-Q22, indicating absence of rearrangement or deletions in the modified BACs. (TIF) [file pgen.1005182.s001.tif]

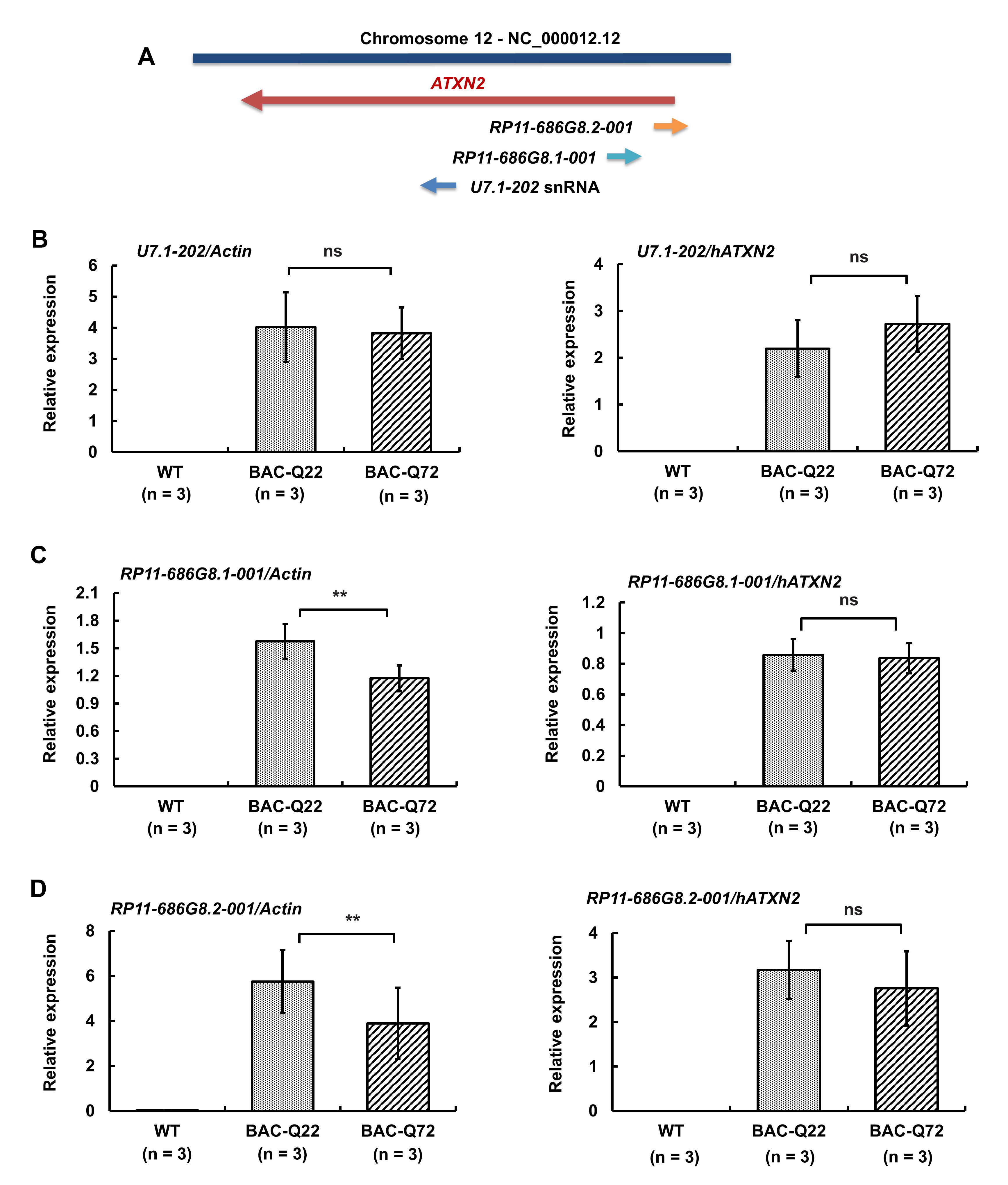

Supplement: S2 Fig — (A) Genomic organization of the human ATXN2 region and intervening genes. (B-D) Relative expression levels of each intervening gene are assessed by qRT-PCR in wild-type, BAC-Q22, and BAC-Q72 cerebella with respect to actin or human ATXN2. U7.1–202 snRNA expression levels do not change significantly between BAC-Q22 and BAC-Q72 animals when normalized to either actin or human ATXN2 (B). Relative expression levels of RP11-686G8.1–001 and RP11-686G8.2–001 are comparable in BAC-Q22 and BAC-Q72 animals when normalized to actin (C, D; left panels). However normalization with human ATXN2 does not result in significant differences between BAC-Q22 and BAC-Q72 animals (C, D; right panels). Three animals from each group were used for these analyses. Data are means ± SD, **p<0.01, Student t-test. (TIF) [file pgen.1005182.s002.tif]

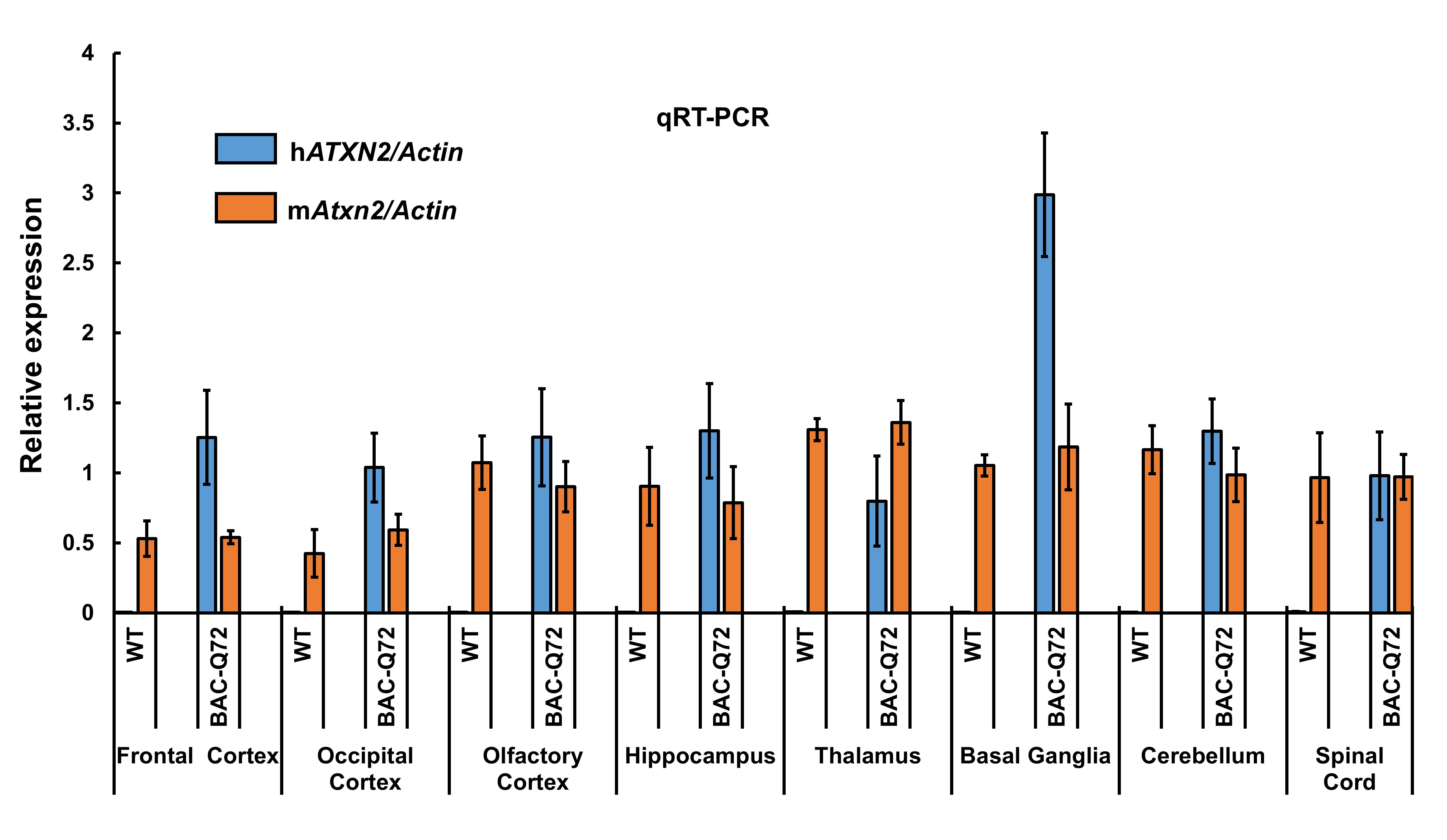

Supplement: S3 Fig — Expression of hATXN2 and mouse Atxn2 mRNAs in different regions of BAC-Q72 and wild-type mouse brains are shown. Quantitative RT-PCR was used to determine transcript levels from RNA isolated from brain sub-regions as indicated in the graph. Three animals per group were used for these analyses. The error bars indicate ± SD. (TIF) [file pgen.1005182.s003.tif]

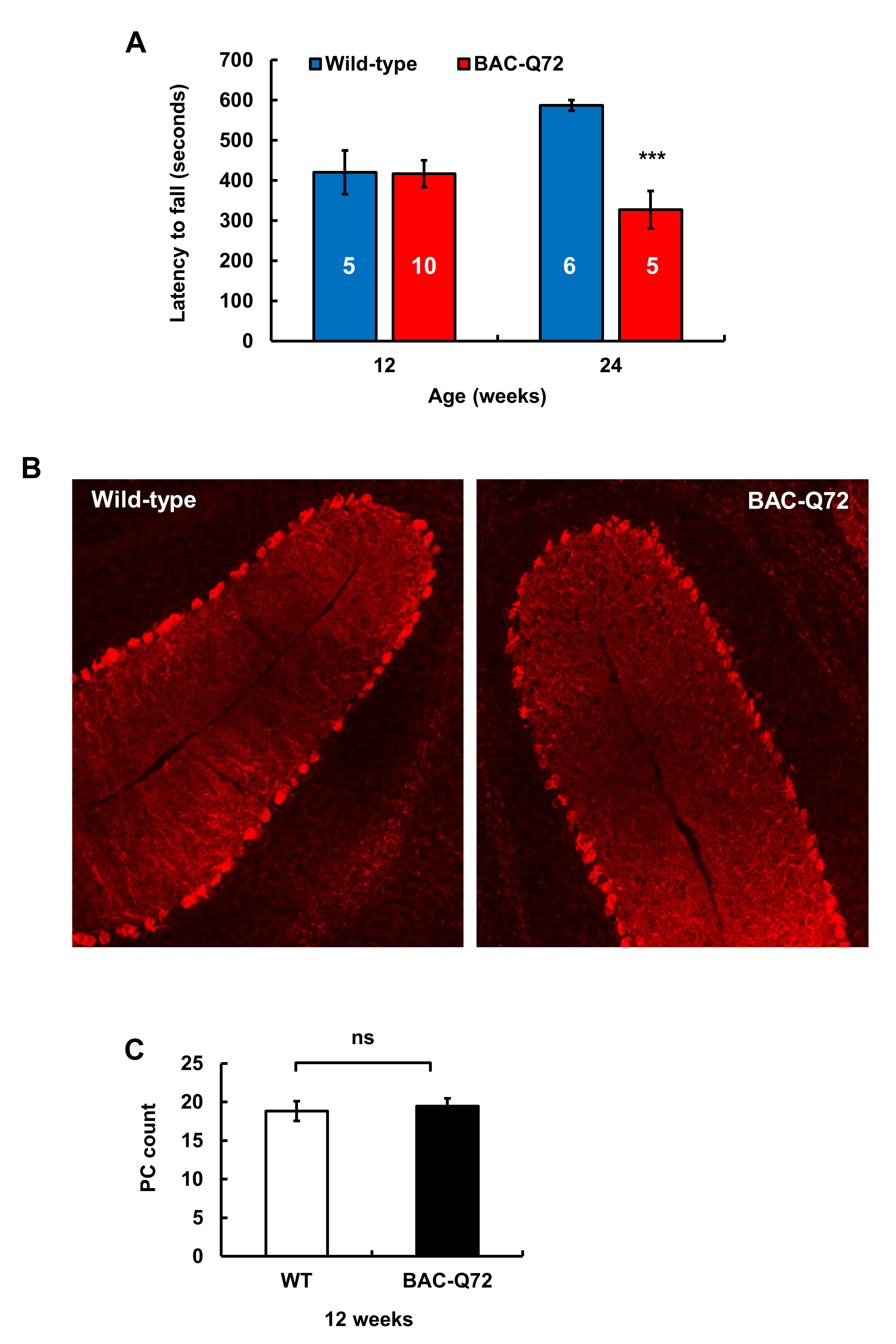

Supplement: S4 Fig — Rotarod performance did not differ significantly in BAC-Q72 mice at 12 weeks of age compared with wild-type littermates in contrast to significantly poorer performance at 24 weeks of age. Data represent the mean ± SEM of three trials on the test day (day 3). Number of animals tested are shown within the bars. Significance was determined using repeated measures ANOVA with post-hoc test correction. ***p<0.001. (B) No morphological changes in the cerebellum of BAC-Q72 mice at 12 weeks of age. Representative micrographs of calbindin-28k immunostaining of PCs in the cerebellum of BAC-Q72 and wild-type littermate are shown. (C) PC counts did not differ significantly in BAC-Q72 mice at 12 weeks of age compared with wild-type littermates. Three animals from each group were used. The data are mean ± SD. (TIF) [file pgen.1005182.s004.tif]

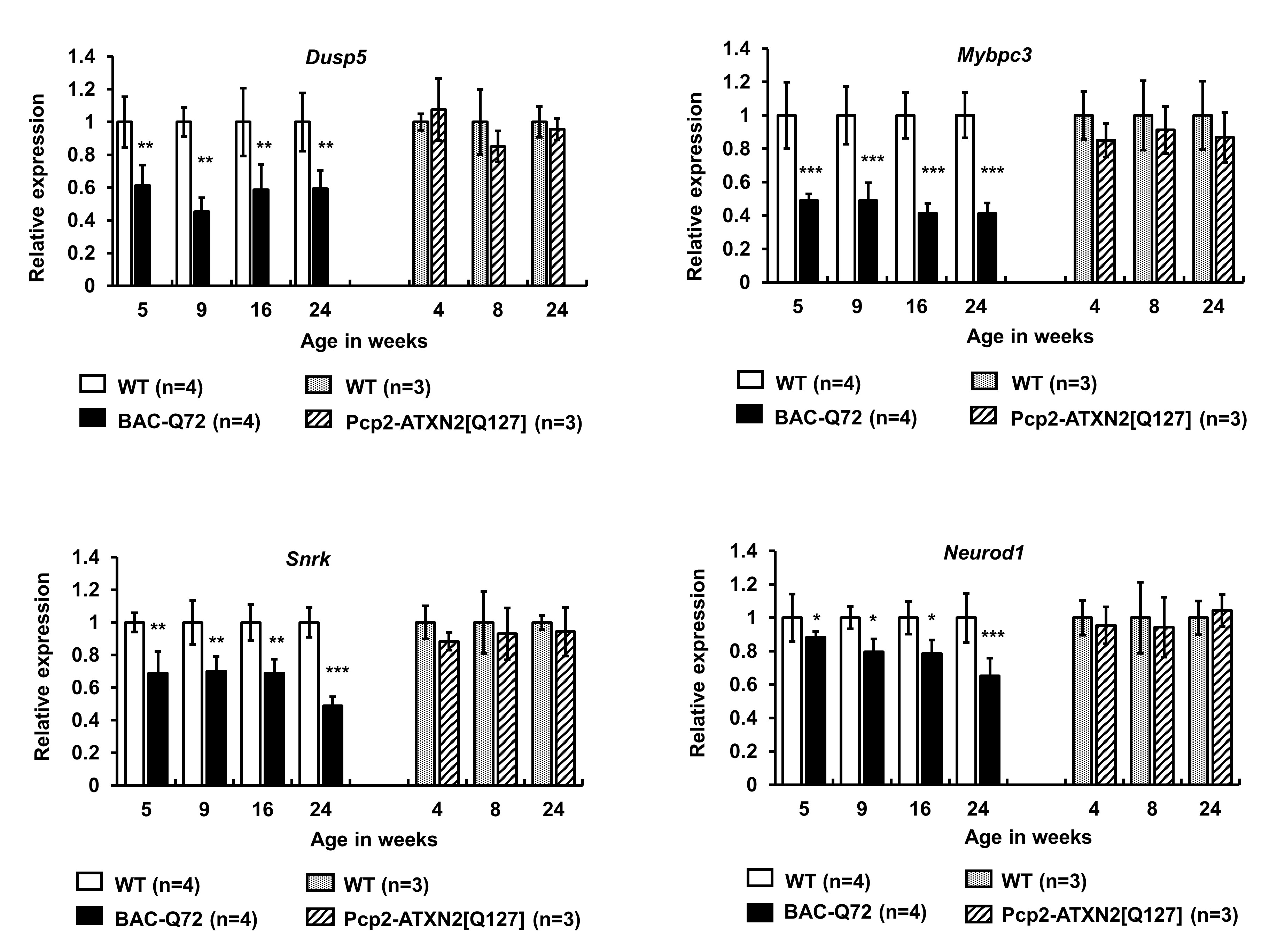

Supplement: S5 Fig — Cerebellar RNAs from BAC-Q72 and Pcp2-ATXN2[Q127] mice, and their respective WT littermates at different ages were used. Genes tested are; Dual specificity phosphatase 5 (Dusp5), Myosin binding protein C, cardiac (Mybpc3), SNF related kinase (Snrk) and Neurogenic differentiation 1 (Neurod1). BAC-Q72 mice show significant and progressive reductions of all transcripts with age when compared with their respective WT littermates. In contrast, all transcripts remained unchanged throughout in Pcp2-ATXN2[Q127] mice compared with their respective WT littermates. Gene expression levels were normalized to beta-actin. n: animal numbers for each genotype and age group are listed in brackets. Data are means ± SD, *p<0.05 **p<0.01, ***p<0.001, Student t-test. (TIF) [file pgen.1005182.s005.tif]

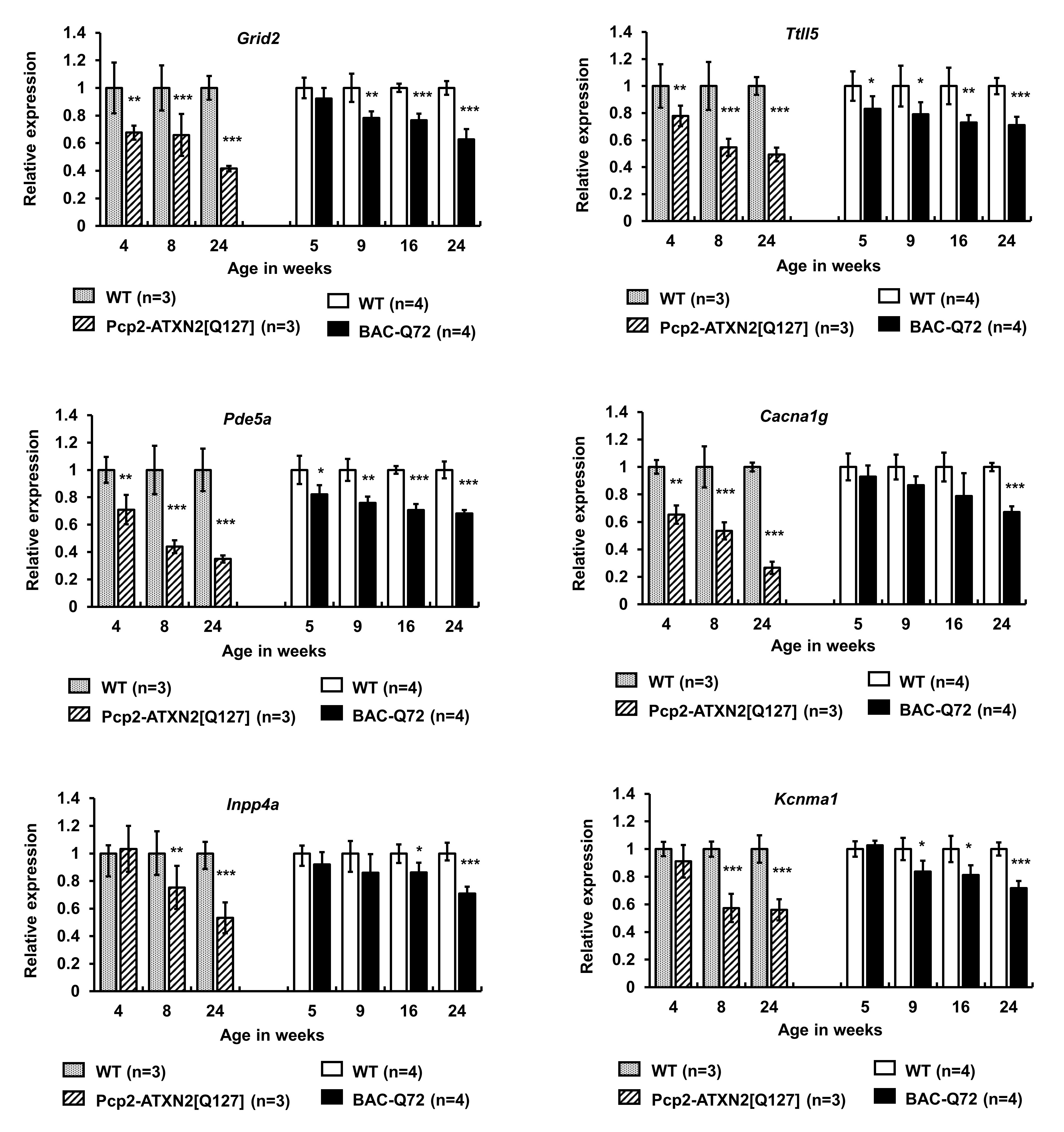

Supplement: S6 Fig — All of the tested transcripts show significant and progressive reduction with age not only in Pcp2-ATXN2[Q127] mice as expected by RNA-seq, but also in BAC-Q72 mice. Only Cacna1g showed late reduction at 24 weeks. Cerebellar RNAs from Pcp2-ATXN2[Q127] and BAC-Q72 mice, and their respective WT littermates were analyzed at the indicated time points. Genes tested are; Grid2, Tubulin tyrosine ligase-like family, member 5 (Ttll5), Phosphodiesterase 5A, cGMP-specific (Pde5a), Calcium channel, voltage-dependent, T type, alpha 1G subunit (Cacna1g), Inositol polyphosphate-4-phosphatase, type I (Inpp4a) and Potassium large conductance calcium-activated channel, subfamily M, alpha member 1 (Kcnma1). Gene expression levels were normalized to beta-actin. n: animal numbers for each genotype and age group are listed in brackets. Data are means ± SD, *p<0.05 **p<0.01, ***p<0.001, Student t-test. (TIF) [file pgen.1005182.s006.tif]

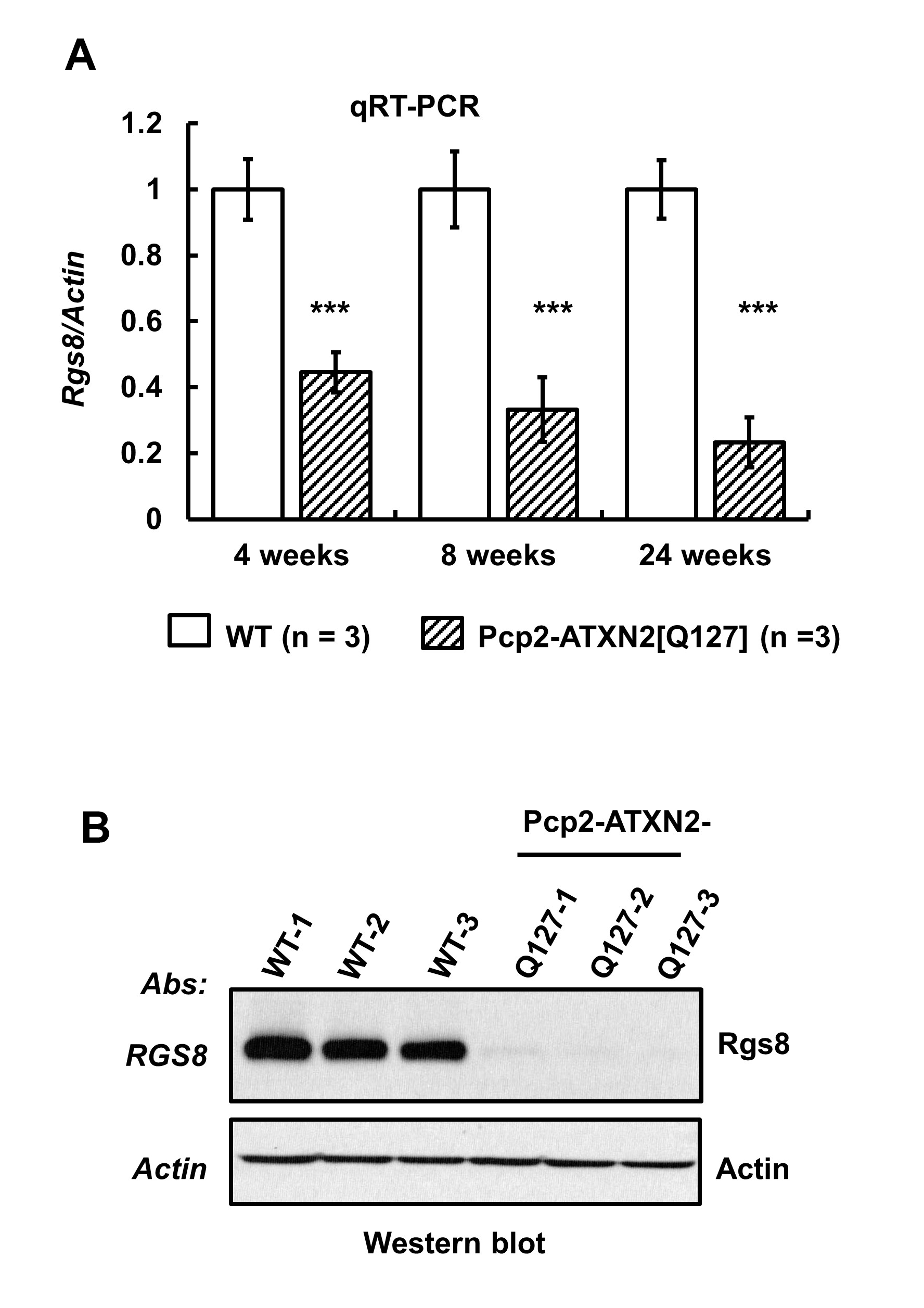

Supplement: S7 Fig — (A) qRT-PCR analyses of cerebellar RNA from wild-type and Pcp2-ATXN2[Q127] mice show significant and progressive reduction of Rgs8 mRNA levels. n: number of animals in each group. The data are means ± SD, ***p<0.001, Student’s t-test. (B) Western blot analyses indicate reduction of Rgs8 steady-state levels in Pcp2-ATXN2 [Q127] mouse cerebella when compared with wild-type mice at 24 weeks of age. Three wild-type and three transgenic animals were tested. The depicted blot is representative of one of 3 independent Western blot experiments. (TIF) [file pgen.1005182.s007.tif]
